# Sustainability and role of plant-based diets in chronic kidney disease prevention and treatment

**DOI:** 10.3389/fphar.2025.1562409

**Published:** 2025-03-31

**Authors:** Giulia Marrone, Manuela Di Lauro, Kevin Cornali, Claudia Masci, Gianluca Vanni, Chiara Vita, Annalisa Noce

**Affiliations:** ^1^ Department of Systems Medicine, University of Rome Tor Vergata, Rome, Italy; ^2^ PhD in Biochemistry and Molecular Biology, University of Rome Tor Vergata, Rome, Italy; ^3^ Breast Unit Policlinico Tor Vergata, Department of Surgical Science, Tor Vergata University, Rome, Italy; ^4^ QuMAP - PIN, University Center “Città di Prato” Educational and Scientific Services for the University of Florence, Prato, Italy; ^5^ UOSD Nephrology and Dialysis, Policlinico Tor Vergata, Rome, Italy

**Keywords:** sustainable diets, natural bioactive compounds, PLADO diet, chronic kidney disease, carbon footprint, Mediterranean diet, renal diets, circular economy

## Abstract

Chronic kidney disease (CKD) affects 10% of the world’s population (namely, 800 million of people) and an increase in CKD prevalence has been observed over the years. This phenomenon in developed countries is related to the spread of chronic degenerative non-communicable diseases (CDNCDs), such as diabetes mellitus, arterial hypertension, obesity, etc., while in low-income to middle-income countries, the CKD prevalence is attributable not only to CDNCDs, but also to infection conditions (like HIV, hepatitis, *etc.*). Another important difference lies in the age of onset of CKD, which is about 20 years lower in developing countries compared to developed ones. Therefore, CKD is becoming a public health problem, requiring preventive and treatment strategies to counteract its spread and to slow its progression. Moreover, the healthcare costs for the CKD management increase as the disease progresses. In this regard, the approach to prevent and reduce the CKD progression involves pharmacological and nutritional treatments (like Mediterranean Diet, MedRen diet, Flexitarian Diet, Vegetarian Diet and Plant-dominant Low Protein Diet) in order to improve the patients’ quality of life and, at the same time, promote the environmental sustainability. Recent studies have highlighted the benefits of these diets not only for individuals, but also for environment. In particular, plant-based diets have increasingly gained an important role in the prevention and management of chronic diseases, including CKD. In fact, recent scientific studies have highlighted how a greater adherence to predominantly plant-based diets, is associated with a lower risk in developing CKD and also in slowing its progression. With regard to environmental sustainability, it is known how our food choices influence the climate crisis, since the food sector contributes for the 25% to the greenhouse gas emissions. Therefore, to reduce the consumption of animal proteins and to replace them with plant-based proteins are key strategies for sustainability and health, also supported by the European policies. In this context, food industries are starting to increase the offer of plant-based products that have similar characteristics, both sensorial and nutritional, to those of animal origin. This innovation, in fact, presents difficulties due to the perception of taste and the organoleptic appearance of these products. An additional challenge concerns the resistance of the traditional food industry and the lack of awareness of the consumer. The paradigm shift is dictated by the obtained benefits for health and for environment. Life cycle assessment studies have compared the land footprint, carbon footprint and blue water footprint of plant-based products with those of animal origin and pointed out the lower environmental impact of the former. In conclusion, the adoption of sustainable food models will slow down the spread of CDNCDs, such as CKD, positively impacting both on human health and on planet, significantly reducing the costs and resources of the National Health Systems, since they absorb up to 70%–80% of the healthcare costs.

## 1 Introduction

Chronic kidney disease (CKD) is a progressive and degenerative disease that affects approximately 10% of the world’s population (10.4% men and 11.8% women), namely, more than 800 million individuals (Mills et al., 2015; Jager et al., 2019). For example, it is estimated that it affects 6%–7% of the Italian population (Ministero della Salute, 2020) and the 11%–12% of the English population (Hounkpatin et al., 2020).

This high incidence is due to the widespread presence of its main risk factors (such as diabetes mellitus, arterial hypertension and obesity, *etc.*) as they could impact on the CKD onset and worsening, and to the world’s population aging (Lv and Zhang, 2019). In detail, diabetes mellitus is considered one of the major causes of CKD, as the presence of chronic hyperglycemia damages the kidney blood vessels, impairing their ability to filter blood. This impairment leads to the diabetic nephropathy, a condition characterized by albuminuria and the progressive reduction in kidney function (Kumar et al., 2023). At the same time, arterial hypertension too is considered one of the major risk factors for CKD development, because of the presence of high blood pressure progressively damages renal microcirculation, consequently reducing the kidney filtration capacity and causing kidney failure (De Bhailis and Kalra, 2022). Moreover, the obesity is able to induce renal dysfunction through direct and indirect mechanisms. The first one is related to the production by adipose tissue of several factors that causes hemodynamic changes, inflammation, oxidative stress, inducing the obesity-related glomerulophaty. The second one is related to obesity complications, such as atherosclerosis, arterial hypertension (due to the hyperactivation of renin-angiotensin-aldosterone system-RAAS), and diabetes mellitus (Nawaz et al., 2023). In low-income to middle-income countries, the CKD prevalence is attributable not only to CDNCDs, but also to infection conditions (like HIV, hepatitis, *etc.*) (George et al., 2017).

Although mortality in the end-stage kidney disease (ESKD), namely, the final stage of CKD, significantly decreased in recent years, CKD continues to represent one of the main causes of death worldwide. In fact, as the Global Burden of Disease (GBD) has estimated, CKD will become the fifth leading cause of death in the world by 2040 (Kovesdy, 2022). This prediction has been formulated according to epidemiological studies that affirms the increasing prevalence of CKD over the years. This enhancement is due to, as already discussed, the aging of the population and the spread of CKD risk factors like diabetes mellitus, arterial hypertension and obesity. Other reasons that will bring to this data is attributable to the underdiagnosis of CKD in the early stages, which contributes to its progression to ESKD and an increased risk of cardiovascular mortality.

The death among CKD patients is mainly related to cardiovascular diseases (Tonelli et al., 2006). CKD patients, especially in the more advanced stages, are at high risk of developing heart failure, arrhythmias, coronary artery disease and sudden cardiac death. The CKD determines the onset of a persistent, chronic and systemic microinflammation that causes the remodeling of blood vessels, stimulating the development of atherosclerotic lesions, the vessels calcifications, the vascular aging, as well as the cardiac muscle fibrosis and heart valves calcifications (Jankowski et al., 2021).

For managing the complex clinical picture of CKD patients, the healthcare costs are substantial. In fact, a CKD patient involves a huge consumption of resources, which increases significantly with the disease progression. These include the direct costs, namely, the managing costs for CKD in the phases of prevention, diagnosis, treatment of the patient (like drugs, medical visits, laboratory tests, instrumental diagnostics, dialytic treatments, hospitalization, *etc.*), and the indirect ones, which refer to the lower productivity of the patient and caregivers, which is further reduced as the disease progresses (Golestaneh et al., 2017; Turchetti et al., 2017).

From a study conducted in 31 Countries, the average costs for the conservative treatment of CKD in the 3a-3b stages, are approximately $3,000–3,500 *per* year, while the costs for the treatment of stage 4 rise up to $5,000 *per* year and they reach more than $8,000 *per* year in the stage 5 (Jha et al., 2023). Moreover, costs for renal replacement treatments (RRTs) increase significantly. On average, hemodialysis costs are $57,000 *per* year, peritoneal dialysis are $49,000 *per* year, and renal transplant approximately are $75,000 in the first year after transplant and $16,000 starting from the second year (Dessi et al., 2014; Jha et al., 2023).

In particular, the Authors observed a four-fold increase in the costs of managing a patient in stage G5, compared to stage G3a (Jha et al., 2023). These healthcare costs increase significantly when the patient is affected by additional CKD-related comorbidities, such as arterial hypertension, CKD mineral bone disorders, *etc.*, (Taccone-Gallucci et al., 2010; Cannata-Andia et al., 2021; Gupta et al., 2023; Li et al., 2023). In detail, the mean annual costs for the management of CKD-related complication are $18,294 for myocardial infarction, $8463 for heart failure, $10,168 for stroke and $5975 for acute kidney injury (Jha et al., 2023). It is estimated that these annual costs are bound to rise, due to the growing CKD incidence and the rampant increase in its risk factors, which accelerate its progression towards the ESKD. In a recent study, a microsimulation model estimated that in 2027, healthcare costs for CKD diagnosis increase up to 9.3% compared to 2022, while those attributed to kidney transplantation up to 10% (Chadban et al., 2024; Tinti et al., 2019).

In general, it has been demonstrated that the healthcare costs of ESKD correspond to 4.7 times the costs for patients with early-stage CKD, highlighting the enormous importance of an early diagnosis. In fact, the earlier the diagnosis CKD is, the sooner patients are taken into care (implementing strategies aimed at slowing the progression of the disease towards the terminal phase). Consequently, the economic savings for their management is greater (Jommi et al., 2018).

As asserted, it is fundamental to adopt preventive strategies aimed at slowing down the CKD progression towards ESKD and at counteracting the onset of CKD-related comorbidities, due to the enormous healthcare costs necessary for CKD treatment. These preventive strategies involve a combination of pharmacological therapies and non-pharmacological adjuvant strategies, which aim at modifying lifestyle habits through the promotion of healthy diets and a regular physical activity (Dabek et al., 2023). In fact, it has been demonstrated that implementing personalized nutritional therapies, starting from the early CKD stages, allows for huge savings in healthcare costs, mainly because it delays the need for RRTs. Specifically, it has been estimated that in Italy the cost of a patient following a nutritional therapy with controlled protein content is around 700 € *per* month; this is a very small cost compared to those estimated for RRTs, which would allow for savings of around 25% of the annual costs necessary for the treatment of CKD patient covered by the NHS (Mennini et al., 2014).

In this context, the nutritional therapies based on the consumption mainly of plant-based foods play a key role. In fact, these types of diets are rich in natural bioactive compounds (NBCs) with numerous beneficial properties for CKD patient’s health. Numerous studies have shown that the consumption of plant-based foods in CKD patients is able to reduce drug treatments, to counteract the development of the main complications of the disease and slow down its progression, and in the meantime to increase the patients survival and improve their quality of life (Joshi et al., 2021; Grazioli et al., 2022; Marrone et al., 2024b).

In this perspective, it is important to underline how these nutritional therapies do not offer important benefits only for the patient himself, but also for the environment. However, it is important to underline that dietary restrictions can be challenging for patients who must follow nutritional plans that limit the intake of protein, sodium, potassium and phosphorus. Symptoms of the disease, such as anorexia, nausea and alterations in taste, can also make it difficult to follow the nutritional plan. Economic factors can also negatively affect adherence to the nutritional plan, since organic foods are more expensive than industrial ones. Finally, managing a personalized diet requires time and the will to be followed, but unfortunately not all patients have the resources to make the prescriptions in practice.

In 2015, the United Nations Organization decided to adopt 17 Sustainable Development Goals (SDGs) that are part of the 2030 Agenda. This is a real call to action that commits all member Countries to achieve a series of goals by 2030, the priority is for: i) wellbeing of the person, ii) protection of the planet and iii) peace and prosperity for man and the planet (2025). The implementation of pant-based diets supports these goals thanks to their sustainability, and thus saving important resources (such as water and land) and reducing greenhouse gas emissions (Kraak and Aschemann-Witzel, 2024).

The aim of this review is to describe the main beneficial effects of plant-based diets with a controlled protein intake for CKD patients and to highlight the importance of their environmental sustainability. In this review, we will analyze the main nutritional diet treatments for CKD patients under conservative therapy, such as Low-Protein diet (LPD), Mediterranean (MD) (MedRen diet and Flexitarian Diet), Plant-Dominant Low-Protein (PLADO) diet and vegetarian diet, giving a particular attention to their environmental sustainability.

## 2 Low-protein diet

In Italy, the origin of LPD can be traced to the Dogma of Giovannetti and Maggiore of the University of Pisa, published on Lancet in 1964 (Giovannetti and Maggiore, 1964). The first half of the 1960s was a historical period in which RRTs were not well developed yet and were scarcely available for CKD patients. For this reason, they often died due to the clinical picture related to terminal uremia.

In developed Countries, the RRTs become available for a greater number of ESKD patients in 1966 (Jacobs, 2009). Therefore, previously, it was necessary to develop a dietary-nutritional therapy (DNT) that allowed CKD patients to survive, slowing down the decline in kidney function and consequently to delay the dialysis treatment. In the study conducted by Giovannetti and Maggiore, eight severe chronic uremic patients have been treated with a LPD, which replaced the protein-deficient diet, characterized by a protein intake of 0.5 g/kg b.w./day and by an adequate caloric intake, up to a 10-month period. The LPD was supplemented with essential amino acids at the dose of 1.74 g per day, fractionated into four or five portions and assumed during the meals, and daily protein intake was achieved by taking a maximum of 2.2 g of high biological value egg proteins *per* day. The Authors concluded that LPD was able to reduce blood urea concentration and the negativity of the nitrogen balance, to maintain for a longer time the residual kidney function and to improve the uremic symptoms. The nitrogen balance became positive or reached the balance when essential amino acids or egg proteins were assumed, without impact significantly on blood urea concentration, thus explaining the re-use of the protein catabolites. Therefore, the LPD is born in 1964 and subsequently it spreads over the years in order to reduce the protein catabolites and to prevent muscle proteolysis. Moreover, in chronic uremia, it has been proved that very low amounts of dietary nitrogen with a high biological value may be sufficient to maintain nitrogen equilibrium (Giovannetti and Maggiore, 1964).

Since the 1980s, the goals of the LPD have changed with the hyperfiltration theory of Barry M. Benner. This theory states that an excessive protein intake causes hyperfiltration and glomerular hypertension, resulting in a faster progression of the kidney damage. Since then, several experimental studies evaluated the LPD effects on preventing glomerular hypertension, on reducing progressive glomerular damage and on the CKD progression (Brenner et al., 1996; Santoro, 2008). LPD reduces the glomerular hyperfiltration through a nephroprotective hemodynamic mechanism, which can be explained by the induction of vascular tone increases of the afferent arteriole, resulting in its vasoconstriction. The synergism of the renin-angiotensin-aldosterone system inhibitors with the low-sodium intake results in a superior additive effect, compared to single pharmacological treatment, through the dilatation of the efferent arteriole and the reduction of intraglomerular pressure and glomerular damage (Kalantar-Zadeh and Fouque, 2017; Cupisti et al., 2020). Moreover, the LPD further reduces the glomerular hyperfiltration and the consequent CKD progression, through these actions: i) the mitigation the mesangial cell signaling, leading to a lower expression of transforming growth factor β and consequently to a reduced interstitial fibrosis; ii) the reduction of nitrogenous compounds, leading to a less production of ammonia and other uremic toxins; iii) the positively impact the gut microbiota, with a lower production of trimethylamine N-Oxide, p-cresol and indoxyl sulfate (namely, the gut-derived uremic toxins); iv) the decrease of acid load; v) the reduction of advanced glycation end products (Kalantar-Zadeh and Fouque, 2017).

The LPD paradigm evolved with the advent of the sodium-glucose-transporter 2 inhibitors (SGLT2i). In fact, in CKD patients, it can be assumed the synergistic effect of LPD and SGLT2i on glomerular hemodynamics. The antiproteinuric and nephroprotective effects are made possible by the fact that both treatments act at the level of the afferent arterial, causing its vasoconstriction and, in CKD patients with type II diabetes mellitus, also at the level of the efferent arterial, increasing its caliber. The result is a reduction in albuminuria (Giannese et al., 2023).

In 1994, Saulo Klahr et al., published on The New England Journal (NEJ) of Medicine the Modification of Diet in Renal Disease Study Group, conducted on 585 patients with GFR comprised between 25 and 55 mL/min/1.73 m^2^. The Authors realized for the first time that the LPD (0.58 g of protein/kg b. w./day) initially reduced GFR, compared to the usual-protein diet (1.3 g of protein/kg b. w./day). They argued that the steeper initial decline probably reflected a hemodynamic response to the reduction in protein intake, rather than an effective CKD progression. As the months passed, patients undergoing LPD experimented a lighter GFR decrease, with a beneficial effect on the CKD progression. After 36 months from baseline, GFR was reduced by 10.3 mL/min/1.73 m^2^ in the LPD group and by 11.2 mL/min/1.73 m^2^ in the usual protein group, without significant differences (Klahr et al., 1994). Although the results were very encouraging, it is necessary to underline the possible limitations of the study, first of all, the difficulty of adhering to dietary recommendations of CKD patients, especially in the long term. In fact, the dietary restrictions imposed can reduce the quality of life of patients, who may feel frustrated or isolated, and this would lead them, if not sufficiently supported, to have poor adherence.

Thirty years later, it was published the effect of dapagliflozin in CKD patients, with or without type 2 diabetes mellitus, with a GFR comprised between 25 and 75 mL/min/1.73 m^2^. The initial hemodynamic effect was the same observed with LPD, while the final outcome was more enhanced. After 30 months from baseline, GFR was reduced by 8.58 mL/min/1.73 m^2^ in the dapagliflozin group and by 11.37 mL/min/1.73 m^2^ in the standard care group, with a significant difference (Heerspink et al., 2020).

In 1998 Bertram L. Kasiske et al., published a meta-analysis that described the effects of dietary protein restriction on the rate of decline in renal function. The Authors analyzed 13 randomized controlled trials, highlighting that a dietary protein restriction reduced the rate of GFR decline by only 0.53 mL/min/year. They concluded that, although LPD delayed the decline of GFR, the relatively weak magnitude of this effect suggests that combined therapies (pharmacological and dietic) are necessary to slow the rate of CKD progression (Kasiske et al., 1998).

In the 21st century, Denis Fouque and Maurice Laville collected ten studies, conducted from 1987 to 2008. A total of 2000 patients were analyzed; in particular, 1002 had received a LPD and 998 a higher protein diet. They showed that in CKD patients, the compliance to the LPD significantly reduced the number of ESKD patients by about 32%, delaying the need to start dialysis treatment (Fouque and Laville, 2009).

In the same century, Giacomo Garibotto et al., confirmed that CKD patients were able to maintain nitrogen balance despite a significantly lower protein intake, but how and to what extent muscle protein metabolism adapts to a LPD was still unexplored. The Authors demonstrated that the LPD (0.55 g of protein/kg b. w./day), compared to the conventional diet (1.1 g of protein/kg b. w./day), induced i) a decrease in muscle protein degradation, ii) the nitrogen balance, iii) no change in muscle protein synthesis, iv) a slight decrease in whole-body protein degradation and v) an increase in the efficiency of muscle protein turnover (Garibotto et al., 2018), thus promoting a physiological body composition in these patients.

The key elements in the success and safety of the LPD are the proper setting of drug therapy by the nephrologists, the proper setting of DNT therapy by the nutritionists and the appropriate adherence to the DNT by the patients. In this regard, in 2018 Cupisti et al. published a consensus document, defining twenty essential points to highlight several relevant aspects of the nutritional approach in patients with a CKD advanced stage. The number five and the number eleven reiterate how an adequate DNT must provide, in addition to the reduction of protein intake, an adequate calorie intake, a reduced intake of sodium, a reduced or controlled intake of phosphorus and potassium and a limitation of the fixed acids load (Cupisti et al., 2018a). Although the leitmotif of the LPD is the reduced protein intake, The National Kidney Foundation’s Kidney Disease Outcomes Quality Initiative (KDOQI) guidelines recommend an energy intake between 25 and 35 kcal/kg b.w./day, adjustable according to age, sex, level of physical activity, body composition and body weight goals. In fact, if patients’ daily caloric requirements are not met, the nitrogen balance becomes negative with protein degradation and loss of lean body mass (Cupisti et al., 2018a; Ikizler et al., 2020). To confirm this, in the study conducted by Nanhui Zhang et al., a protein-controlled DNT, characterized by a reduced carbohydrate-derived caloric intake (150 g carbohydrates/day vs. 264 g carbohydrates/day), increases the risk of all-causes mortality (Zhang et al., 2022). Regarding micronutrients, special attention should be paid to an intake less than 3,000 mg/day of potassium, (Cupisti et al., 2018b), 700 mg/day of phosphorus (Cupisti et al., 2018a) and 2.3 g/day of sodium (Ikizler et al., 2020).

The statement number twelve of the consensus document confirms that LPD may delay the need for RRT, while the number sixteen states that an appropriate DNT allows to contain the costs and the resources by National Healthcare Systems, for the clinical management of ESKD patients (Cupisti et al., 2018a). However, patient eligibility for the LPD remains at the discretion of the nephrologists (Ikizler et al., 2020; Kidney Disease: Improving Global Outcomes, 2024). In this regard, the recent publication of the NEJ about an interactive clinical decision inherent to a patient eligible for the LPD collected 2,628 total responses: 38% recommended it, while 61% did not (Chang et al., 2024).

Over time, LPD has also been positively influenced by issues, related to environmental sustainability and how plant-based foods are beneficial to the organism, so much so that predominantly plant-based declinations of LPD were born (Kalantar-Zadeh et al., 2020).

## 3 Possible advantages of plant-based diets in chronic kidney disease

In recent years, increasing evidence has highlighted the advantages of plant-based diets in preventing and managing lifestyle-related diseases, including CKD (Katz and Meller, 2014). Currently, many studies suggest a lot of benefits in using this dietary approach for treating CKD and its common comorbidities (De Angelis et al., 2007; Joshi et al., 2020).

Enhancing the nutrient profile of patients’ diets by incorporating more plant-based foods and reducing animal protein may help to decrease the reliance on nephroprotective medications, to counteract CKD complications and potentially to slow the disease progression, thus improving patient survival rates (Patel et al., 2012; Haghighatdoost et al., 2017; Haring et al., 2017).

Among the main criticisms and objections to these diets, there are the hyperkalemia and the protein deficiency. By thoroughly evaluating these potential risks, it emerges that these events may be less severe than believed, while the benefits are substantial (Babich et al., 2023). Overall, the risk-benefit ratio seems to increasingly support the broader adoption of plant-based diets.

More generally, the plant-based diets, if not well balanced, may be deficient in essential amino acids. In order to avoid this condition, it is essential to combine different protein sources (e.g., legumes and cereals). Iron intake must also be closely monitored as iron of plant origin (non-heme iron) is less bioavailable than animal iron. In order to prevent possible deficiencies, it is advisable to take iron-rich foods in conjunction with a source of vitamin C to improve its absorption (Marrone et al., 2021).

Omega-3 fatty acids, particularly eicosapentaenoic acid (EPA) and docosahexaenoic acid (DHA), are mainly contained in the fish and they may be deficient in plant-based diets. In this case, it would be advisable to supplement these nutrients through specific food supplements (Lane et al., 2022). Zinc and iodine may also be deficient as the former is less bioavailable in plants, while the latter may be deficient if iodized salt is not consumed (Bakaloudi et al., 2021).

Another possible limitation of plant-based diets may be a greater tendency to consume refined carbohydrates and sugars than proteins, posing a greater risk for metabolic alterations (Key et al., 2022).

In this context, the dual aspect of plant-based diets in CKD is of notable interest. On one hand, it can serve as an excellent strategy to slow the disease progression, while on the other, it may be considered for the prevention of the disease’s onset (Chauveau et al., 2019; Zarantonello and Brunori, 2023). The Tehran Lipid and Glucose Study (TLGS) (Azizi, 2018) and the Multi-Ethnic Study of Atherosclerosis (MESA) (Blaha and DeFilippis, 2021) are the two main cross-sectional studies that investigated the incidence of CKD according to the kind of the protein consumed. TLGS study found that among the 5.000 participants, those who consumed a higher amount of plant proteins showed a 30% lower risk to develop CKD, compared to those who consumed less plant proteins. In the MESA, the Authors pointed out that those who consumed a higher proportion of whole grains, fruits, vegetables, and low-fat dairy products showed a lower urinary albumin-to-creatinine ratio. However, these interesting studies should be confirmed by other clinical trials conducted on higher number of patients and with a longer observational period.

Regarding pre-existing CKD conditions, the Nurses’ Health Study (NHS) showed the potential role of plant-based diets in the secondary prevention.

**FIGURE 1:**
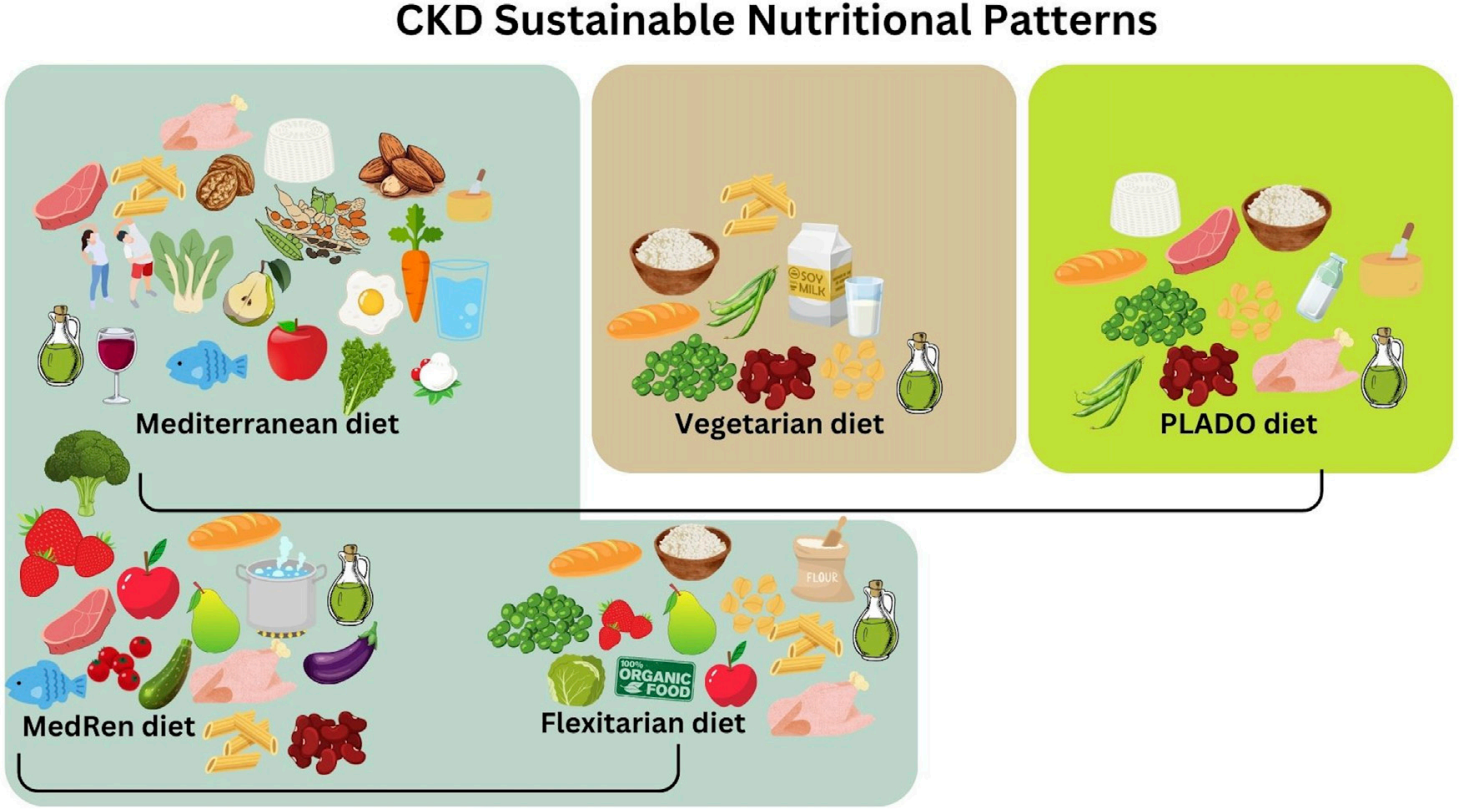
Possible sustainable nutritional patterns in chronic kidney disease (CKD) patients under conservative therapy.

Plant-based foods are recognized as a key part of a healthy diet across a broad range of eating styles. The “common denominator” of all plant-based diets is the prevalence of plant-based proteins within their eating patterns. Among them, the most significant plant-based dietary patterns are the MD, and its adaptations as the MedRen diet and the Flexitarian diet, the Vegetarian diet and the PLADO diet (Figure 1).

### 3.1 Mediterranean diet

The MD is not just a dietary pattern, typical of countries along the coast of the Mediterranean Sea, including Greece, Italy, Spain, and nations of the Middle East. The MD is rather a real lifestyle that in 2010 was included in the list of the “Intangible Cultural Heritage of Humanity” of United Nations Educational, Scientific and Cultural Organization (UNESCO), which describes it as “a set of skills, knowledge, rituals, symbols and traditions concerning crops, harvesting, fishing, animal husbandry, conservation, processing, cooking, and particularly the sharing and consumption of food” (Pett et al., 2017).

The concept of the “Mediterranean diet” was born from the intuition of the American physiologist Ancel Keys, who in the mid-twentieth century tried to understand the reasons for the lower cardiovascular disease incidence rate in the Mediterranean countries. The first study that investigated the beneficial effects of the MD conducted on a global scale, was the Seven Country Study (SCS), which involved researchers from seven countries (United States, Finland, Holland, Italy, Greece, Former Yugoslavia and Japan) with the aim of exploring in detail the associations between eating habits and some risk factors with the rates of cardiovascular diseases (Keys et al., 1984; Pett et al., 2017). The study, after 25 years (1958–1983) of data collection from 16 cohorts of middle-aged men, has demonstrated, for the first time, that high serum cholesterol levels, high blood pressure levels, diabetes mellitus and cigarette smoking are the main universal risk factors for cardiovascular diseases. Furthermore, a higher intake of animal foods, rich in saturated fats and animal proteins (excluding fish) and of simple sugars was associated with a higher rate of cardiovascular mortality; while, a higher intake of foods rich in flavonoids, therefore plant-based foods (such as fruit, vegetables and legumes), was inversely associated, in a statistically significant manner, with cardiovascular mortality rates (Keys et al., 1984; Menotti and Puddu, 2015; Blackburn, 2017).

The MD as a dietary model is characterized by a rich consumption of plant-based foods (cereals, legumes, dried fruit, fresh fruit, vegetables, *etc.*), a moderate consumption of fish and seafoods products, eggs, white meat and dairy products and a low consumption of red and processed meat. The MD also includes a moderate consumption of alcohol (red wine, to be consumed only during meals) and the use of extra virgin olive oil as the main source of vegetable fats (Bach-Faig et al., 2011).

Numerous studies suggest that the MD principles are able to reduce the risk not only of cardiovascular diseases but also of others chronic-degenerative diseases, including CKD (Riboli and Kaaks, 1997; Palli et al., 2003; Cicero et al., 2008; Martinez-Gonzalez et al., 2015).

In literature, it has been reported that the MD, in the early CKD stages, exerts numerous effects, including the reduction of cardiovascular risk factors, the decrease of chronic systemic micro-inflammation and the oxidative stress, the improvement of the lipid profile, the slowing of the progression towards ESKD, the improvement of blood pressure values, and the positive modulation of the gut microbiota (Chauveau et al., 2018; Cigarran Guldris et al., 2022; Perez-Torres et al., 2022).

As a proof, the study conducted by De Lorenzo et al. demonstrated the potential benefits of the Italian Mediterranean Organic Diet (IMOD) in nephropathic patients. In detail, 50 CKD male patients with stage 2–3 (according to the 2013 K-DIGO guidelines (Lamb et al., 2013), after 14 days of MD with organic products, showed an improvement in homocysteine, phosphorus, blood glucose, lipid profile, and microalbuminuria. Moreover, the results highlighted an amelioration of the body composition, measured with Dual-X absorptiometry (DXA). In detail, the Authors observed an increase in the lean body mass of CKD patients as well as a statistically significant reduction in fat mass in both kilograms and percentage (De Lorenzo et al., 2010).

Among natural bioactive compounds, those that mostly characterize the MD are the polyphenols contained in foods of plant origin and particularly in extra virgin olive oil (EVOO). Of particular importance among these are hydroxytyrosol, oleuropein and oleocanthal (Jimenez-Lopez et al., 2020). Numerous scientific studies have shown their beneficial effects on health as they are able to exert powerful anti-inflammatory actions. Therefore, daily consumption of an EVOO can counteract the microinflammatory environment that promotes the progression of CKD (Noce et al., 2021b).

However, it is worth noting that the NBCs in the EVOO are also able to induce cardiovascular protection through the reduction of oxidative stress and to improve purine and lipid metabolisms and the body composition parameters in nephropathic patients (Romani et al., 2020a; Marrone et al., 2022). The main variations of the MD are the MedRen diet and the Flexitarian diet.

#### 3.1.1 MedRen diet

The Mediterranean Renal Diet (MedRen diet) is a modified version of the MD, characterized by a quantitative decrease in the recommended daily allowances (RDA) for protein, salt and phosphate, compared to the general population’s standards. In particular, it should be formulated in order to achieve an amount of 0.8 g of proteins/day, 6 g of salt/day and less than 800 mg of phosphorus/day (Naber and Purohit, 2021). This diet is outlined by the presence of a greater number of plant-based foods that allow patients to assume a higher proportion of alkalizing compounds, fiber, and polyunsaturated fatty acids, than foods of animal origin (D’Alessandro et al., 2023). This dietary pattern also turns out to be easily accepted by patients and thus favoring a higher patients’ adherence to the personalized nutritional plan.

The “strength” of this diet is that following the traditional MD and only making minor “adjustments” it preserves the renal function and supports the metabolic patterns, which are often compromised in mild-to-moderate CKD (Narasaki and Rhee, 2020; Noce et al., 2021c; Babich et al., 2023). The main recommendations include: i) to prefer white meat instead of red meat, ii) to increase the consumption of vegetable proteins as legumes, iii) to define portions and cooking methods according to the patient’s needs/requirements (D’Alessandro et al., 2023). For example, in case of hyperkalemia it is advisable to select fruit types with less potassium content and eat at maximum two portions of vegetables *per* day, properly boiled before consumption. Hard cheeses should be avoided (maximum two teaspoons of Parmesan *per* day) and fresh cheeses (such as cow ricotta cheese) once every 7–10 days. Hydrate in the correct way, introducing a share of water equal to the sense of thirst. Increase, if necessary, the amount of fluids with infusions and/or hot drinks, paying attention to possible drug interactions.

An interesting study conducted by Cupisti et al. (2015) showed that, after 6 months of nutritional treatment, a group of 93 stage 3 CKD patients compared to 223 stage 3 CKD patients (that did not follow any nutritional treatment), showed lower values of azotemia, phosphorus and parathyroid hormone (PTH), suggesting its potential role also in the management of CKD-mineral bone disorders.

In this context, the Mediterranean Renal Diet can be considered as a “change” from a healthy eating pattern to a diet “appropriate” for mild-to-moderate CKD. This approach, lending itself particularly well to being followed daily by patients, yields excellent results in terms of long-term adherence to the nutritional plan (D'Alessandro et al., 2023).

#### 3.1.2 Flexitarian diet

The world of plant-based diets is heterogeneous. Current definitions range from the complete exclusion of all animal products, to the partial inclusion of fish, poultry and yogurt, thus effectively reducing processed foods and saturated fats. In this perspective, a plant-based diet can be declined toward different patterns that do not necessarily frame the vegan or vegetarian diet (Storz, 2022).

The Eat-Lancet Commission describes the flexitarian diet as a plant-based pattern, which favors plant-based foods but may occasionally include small portions of fish, meat and dairy products (Willett et al., 2019; EAT Lancet, 2025).

In more details, Spiringmann et al., in the journal Lancet Planet Health define the flexitarian diet as a dietary pattern that contains no processed meat, modest portions of red meat and sugar, moderate amounts of poultry, dairy, eggs and fish and high amounts of fruits, vegetables and legumes (Springmann et al., 2018).

A distinctive feature of the flexitarian diet is that it takes into consideration the ethical aspects of the food chain. In particular, this dietary pattern is aimed at improving animal welfare, avoiding agricultural intensification, inefficient production of foods, greenhouse gas emissions and issues about dietary and public health problems (including zoonoses and veterinary antibiotic use) (van der Weele et al., 2019). Moreover, the flexitarian diet deters long-term consumption of red and processed meats because of its association with increased risks of mortality, cardiovascular diseases, and cancer (Pighin et al., 2016). An additional aspect that distinguishes the flexitarian diet is that as well as encouraging the consumption of foods of plant origin, it promotes foods of local tradition and suggests their consumption according to its seasonality For these reasons, in the CKD scenario, the flexitarian diet could be a useful dietary model, both in its prevention and in the management of those patients, who have already developed CKD. In fact, this diet implies not to completely exclude foods of animal origin but rather to consider their potential benefits on the health maintenance, paying attention to their qualities and quantities.

A Finnish study suggests that in a flexitarian pattern, the protein intake should be no more than 30% from animal sources and that the main share of calories should come from foods such as legumes, whole grains, fruits and vegetables (Pellinen et al., 2022), indications that fit well with the preventive strategies currently used to combat CKD onset.

A recent study conducted in 2024 by Bruns et al. (2024) evaluated the cardiovascular risk associated with the flexitarian diet compared with the omnivore and vegetarian diets. Their results showed in those who followed a flexitarian diet, better metabolic health (assessed by MetS-score), better body mass index and in particular, waist circumference and better pulse wave velocity, compared with both vegetarians and omnivores. This result lends itself to support the hypothesis that the flexitarian diet is cardioprotective, demonstrating that lower consumption of red and processed meats and the promotion of the consumption of plant-based foods, obtained from a sustainable supply chain, can significantly reduce the risk of developing cardiovascular diseases.

Currently, there are no studies conducted on CKD patients and flexitarian dietary nutritional therapy in the literature. We can speculate that its particular composition may exert a significant impact, due to what was discussed earlier, in terms of prevention and improved therapeutic management of the nephropathic patients. In fact, its peculiar characteristic of not completely excluding foods of animal origin, but rather of selecting their quantity and quality, allows the body to get its fair share NBCs such as vitamins (B12), minerals (zinc, iron, calcium), peptides (essential amino acids) or fatty acids (like omega-3) that combine themselves with those found in plant origin foods (Pogorzelska-Nowicka et al., 2018).

### 3.2 Vegetarian diet

Vegetarian diets are nutritional regimes characterized by a greater consumption of plant-based foods, compared to those of animal origin. There are different types of vegetarian diets: the vegan diet, which completely excludes all foods of animal origin; the lacto-ovo vegetarian diet, which includes the consumption of eggs and dairy products; the pescatarian diet, which allows the consumption of fish and fish products; and, finally, the so-called “flexible” vegetarian diet, which includes moderate consumption of poultry (Gluba-Brzozka et al., 2017). Regardless of the type, vegetarian diets are characterized by a large consumption of legumes, fresh fruits and vegetables, excluding the consumption of ultra-processed foods. These types of diets, therefore, are a rich source of dietary fibers, vitamins and phytochemicals (Narasaki et al., 2023).

A vegetarian diet has been shown to improve renal filtration in CKD patients and reduce the major risk factors for the disease onset, such as body mass index, blood pressure, fasting glucose, low density lipoprotein-cholesterol and triglycerides levels (Dinu et al., 2017; Swiatek et al., 2023).

One of the main points of debate, in CKD patients, regarding the vegetarian diets, is the serum potassium intake. It should be underlined that serum potassium levels can be elevated in patients with a reduced kidney function mainly after the consumption of foods with potassium-based additives or with a highly concentrated potassium content (such as juices, dried fruit, or purees). Instead, fresh plant-based foods may have properties that help to reduce potassium retention, such as their alkalizing effects, the limited bioavailability of potassium, and the role of dietary fiber in organic plant foods, in promoting potassium excretion through the colon. Additionally, by following the traditional low-potassium “renal diet,” CKD patients may miss out on many of the benefits that plant foods offer. For this reason, the latest dietary recommendations for kidney health advocate for patient-focused recipes centered around plant-based foods without restricting them (Babich et al., 2023).

The vegetarian diet specifically designed for CKD patients is the “renal vegan diet”. The vegan diet is characterized by a protein intake of 0.7 g/kg b. w/day and provides adequate support of essential amino acids derived from a close combination of cereals and legumes (Barsotti et al., 1996). The vegan diet results in more favorable outcomes, including reduced net acid production, a stronger anti-proteinuric effect, and lower phosphorus intestinal absorption, compared to omnivorous diet with the same protein content (Cupisti and Kalantar-Zadeh, 2013).

Fresh plant-based foods, highly recommended in vegetarian diets, are rich in NBCs with numerous beneficial properties for CKD patients. Among these, the most abundant are polyphenols that positively impact on the slowing of the CKD progression through different mechanisms: by means of an antioxidant action, as scavenger of reactive oxygen species (ROS) and as supporter of the natural antioxidant defenses; by means of an anti-inflammatory action, with the reduction of pro-inflammatory cytokines production; by means of the protection from cells and tissues damage and the attenuation of endothelial dysfunction, typical of CKD patients (Cho et al., 2018; Grazioli et al., 2021; Natesan and Kim, 2025).

### 3.3 Plant-dominant low-protein diet

In 2020, Kalantar-Zadeh delineated a new type of diet suitable for CKD patients, characterized by a prevalent consumption of plant-based proteins (i.e., from legumes) to the detriment of those of animal origin. The Authors defined this diet as plant-dominant LPD, or PLADO diet (Kalantar-Zadeh et al., 2020). The PLADO diet is characterized by a protein intake equal to 0.6–0.8 g/kg/day, of which at least 50% from plant-based sources, thus avoiding ultra-processed foods. The PLADO diet also provides an adequate dietary energy intake (i.e. 30–35 Kcal/kg/day), a low sodium intake (i.e., <3 g/day) and a high fiber intake (at least 25–30 g/day) (Kalantar-Zadeh et al., 2020).

The benefits that this type of diet exerts on slowing the progression of renal damage are carried out through numerous mechanisms. First of all, there is a reduction in glomerular hyperfiltration. Moreover, an amplification of the therapeutic effect of RAAS and SGLT2 inhibitors, with the reduction of intraglomerular pressure, has been demonstrated (Koppe and Fouque, 2019). Furthermore, the reduction of nitrogenous compounds intake, leads to a lower production of urea and uremic toxins, so improving the control of uremia and delaying the start of dialysis.

The lower bioavailability of phosphorus in vegetal proteins, compared to those of animal origin, allows a better control of serum phosphorus. The higher fibers intake, in addition to reducing the acid load of the diet (Rodrigues Neto Angeloco et al., 2018), is able to reduce the production of advanced glycation end-products (AGEs), which intervene in the renal damage progression (Demirci et al., 2019). An increased consumption of plant-based foods leads to a higher production of antioxidant and anti-inflammatory molecules, thus reducing the chronic micro-inflammation and oxidative stress, typical of CKD patients (Ko and Kalantar-Zadeh, 2021; Kalantar-Zadeh et al., 2022; Sakaguchi et al., 2023).

Finally, the consumption of plant-based foods, rich in fiber, allows to the positive modulation of gut microbiota. Recent studies have shown that CKD patients are characterized by gut dysbiosis. This is typified by an increase in bacteria responsible for proteolytic fermentation, which leads to the increased production of gut-derived uremic toxins (such as indoxyl sulfate, oxidized trimethylamine and p-cresyl sulfate) and a reduction in bacteria responsible for saccharolytic fermentation, which, instead, produce beneficial molecules, including short-chain fatty acids (SCFAs) (Noce et al., 2022). The former is able to impair the barrier function and deplete the intestinal tight junctions, allowing intestinal bacteria to reach the bloodstream and exacerbate inflammation and multi-organ damages. The latter, on the other hand, help maintaining the intestinal health, ensuring good functionality, exerting an anti-inflammatory action and positively modulating the immune system (Lau et al., 2015; Lau et al., 2018; Noce et al., 2022). The consumption of foods rich in fiber, such as plant-based foods, in CKD patients, is able to restore the balance of the gut microbiota, leading to an increase in saccharolytic bacterial species (such as the Prevotellaceae and Lactobacillaceae families) and a concomitant reduction in proteolytic bacterial species (such as the Enterobacteriaceae family) (Wiese et al., 2021; Koppe and Soulage, 2022).

The main source of vegetable protein in the PLADO diet is represented by legumes. The latters, in addition to being an excellent source of protein (mainly rich in lysine), show a high quality of complex carbohydrates and soluble fibers (with a low glycemic index), a low-fat content (mainly represented by linoleic acid) and an excellent content of vitamins (A, E, and B) and minerals (poor in sodium and rich in potassium, zinc, calcium and iron). Moreover, legumes are an important source of phytochemicals that exert numerous beneficial effects on health, including renal ones. In particular, they are phytosterols, tocopherols, alkaloids, carotenoids, flavonol, flavone and isoflavone (Tor-Roca et al., 2020; Yin et al., 2023).

## 4 Plant-based diets sustainability

Our food choices can influence the course of the climate crisis, especially with regard to the issue of global warming, as the food sector is responsible for approximately 25% of greenhouse gas emissions worldwide (FAO, 2019). To address current sustainability challenges and reduce greenhouse gas emissions, the food system as a whole must also be changed by acting both on waste reduction and on changes in dietary habits, replacing animal proteins with plant based-proteins. At this regard, the current political agendas of the European Union (EU) are focused on the increase consumption of plant-based proteins (EuropeanCommission, 2022).

**FIGURE 2:**
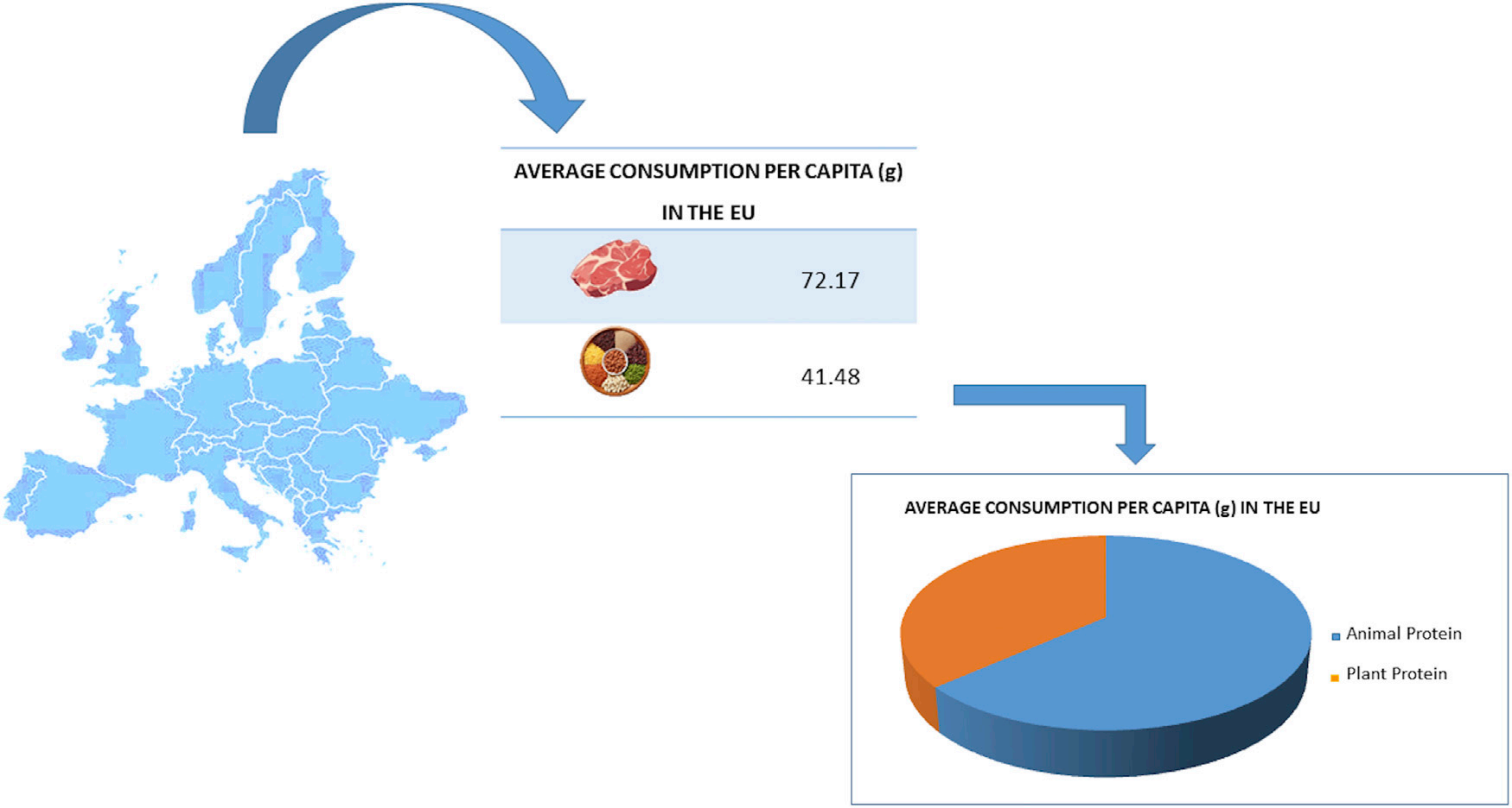
The average consumption *per capita* (g) of different protein sources in the European Union (EU).

One of the most frequently proposed dietary modification measures is to reduce the consumption of foods of animal origin, especially meat, together with the advice to increase the consumption of plant-based foods. The current scenario in the EU sees an average meat consumption *per capita* of 72,17 g, while plant protein provide an average consumption of 41,48 g (FAO, 2023) (Figure 2).

In recent years, there has been an emerging trend towards a greater consumption of plant-based proteins, such as plant-based hamburgers, milk or yogurt, as healthy substitutes for animal-based products (Jaeger et al., 2023).

Nowadays, the demand for sustainable, nutritious and healthful foods has greatly increased and the market has been enriched with processed plant-based products that are alternatives to animal products, especially meat products. The goal of the food industries is to obtain foods with sensory, structural and nutritional characteristics similar to those of animal origin.

At this regard, comparative studies of life cycle assessment (LCA) were applied to both plant and animal proteins. LCA is a method used to evaluate the impact of a product on the environment, taking into account all inputs and outputs in the various phases of the product’s life cycle, that is from the extraction of raw materials to the production, transport, use and disposal (ISO 14040:2006).

The evaluation parameters for the comparison between proteins of animal and plant origin were based on land footprints, carbon footprints and blue water footprints (CarbonTrust, 2021). Comparative carbon footprint data between animal and plant proteins clearly show that plant proteins tend to have a lower environmental impact than animal proteins.

Poore and Nemecek (2018) were the first to conduct a comprehensive analysis of the ecological footprint of various foods, comparing animal and plant proteins in detail. The results are summarized in Tables 1, 2.

**TABLE 1:** kg CO_2_-equivalents per kg of product for animal proteins.

	kg CO_2_-equivalents for kg of product
Beef	60
Pork meat	12
Chicken	6
Fish (e.g., salmon)	4.5
Dairy products (milk and cheese)	7.5

**TABLE 2:** kg CO_2_-equivalents per kg for plant-based proteins.

	kg CO_2_-equivalents for kg of proteins
Soy (protein isolate)	1.9
Lentils	0.9
Peas	1.3
Rice (protein isolate)	2.5
Wheat (protein isolate)	2.5

As shown in the data, plant-based proteins have a significantly lower environmental impact than animal-based proteins, especially when compared to the red meat (e.g., beef), which owns the highest carbon footprint. On the contrary, plant-based proteins such as soy, peas and lentils are particularly sustainable, with a carbon footprint ranging from 0.9 to 2.5 kg CO_2_-equivalents per kg of proteins.

Plant-based meat alternatives (such as pea-based veggie burgers) are significantly less impactful than traditional meat, making them an attractive option for reducing greenhouse gas emissions in the environment. The sustainability of some plant-based foods is twofold. For example, legumes, present a very low greenhouse gas (GHG) intensity (known as “carbon footprint”) per unit of nutritional density compared to other foods (Williams et al., 2020). Another very important aspect relating to the sustainability of legumes cultivation concerns their characteristic of being nitrogen-fixing soil. In fact, their cultivation does not require the application of nitrogen fertilizers, by virtue of their ability to biologically fix atmospheric nitrogen, improving the structure and presence of micronutrients in the soil (Meena et al., 2017).

In this perspective, consumers prefer plant protein-based products because of their potential health benefit, while can be observed negative health effects related to diets high in animal proteins.

Moreover, an increased focus on sustainable foods and ethical issues regards the treatment of the animals (Hertzler et al., 2020). We need to underline, it was estimated that globally the food production is the largest contributor to biodiversity loss and is responsible for 80% of deforestation, >70% of freshwater use and 30% of GHG emissions (Nelson et al., 2016).

In the 2010, the FAO defined as sustainable diet “diets with low environmental impacts which contribute to food and nutrition security and to healthy life for present and future generation”. In particular, sustainable diets should: i) have a low environmental impact (minimizing the use of natural resources, thereby reducing the strain on ecosystems and protecting biodiversity; promoting food and nutritional safety); ii) ensure access to sufficient, safe, and nutritious food sources for a healthy living now and in the future; iii) be culturally acceptable and accessible (align with different cultural norms and practices, remaining at the same time, economically and physically available to all); iv) be economically fair and affordable (support equitable access to food, while being cost-effective for producers and consumers) (Burlingame et al., 2011).

These advantages make plant-based proteins a key component in promoting sustainable diets, as they align with the FAO’s goal which tends to reduce environmental impact and to support food safety and nutritional quality (Medina-Vera et al., 2024).

The shift towards plant-based foods reflects the growing awareness for environmental sustainability, animal welfare and human health benefits. Plant-based foods are rich in dietary fibers and natural bioactive compounds, which contribute to increase their nutritional value. Their production and development copes with sustainable practices, reducing the environmental impact associated with animal-based food production.

This trend has driven to some innovations in the food processing technologies in order to enhance the quality, safety, and appeal of the plant-based products. These advancements focus on improving texture, flavour and nutritional properties. The increasing demand for plant-based alternatives underscores the need of a ceaseless research and development in this field.

In terms of ecological and environmental protection, the development and the utilization of plant protein-based products go along with the goals of resources recycling. Plant-based foods production generally requires fewer resources compared to those required for the animal products, making it in accordance with the principles of the low-carbon sustainable development. The production of plant-based products is characterized by a lower carbon footprint, so it requires less land, water, and energy compared to the production of animal-based products. This makes them an excellent choice for supporting a low-carbon sustainable development.

**FIGURE 3:**
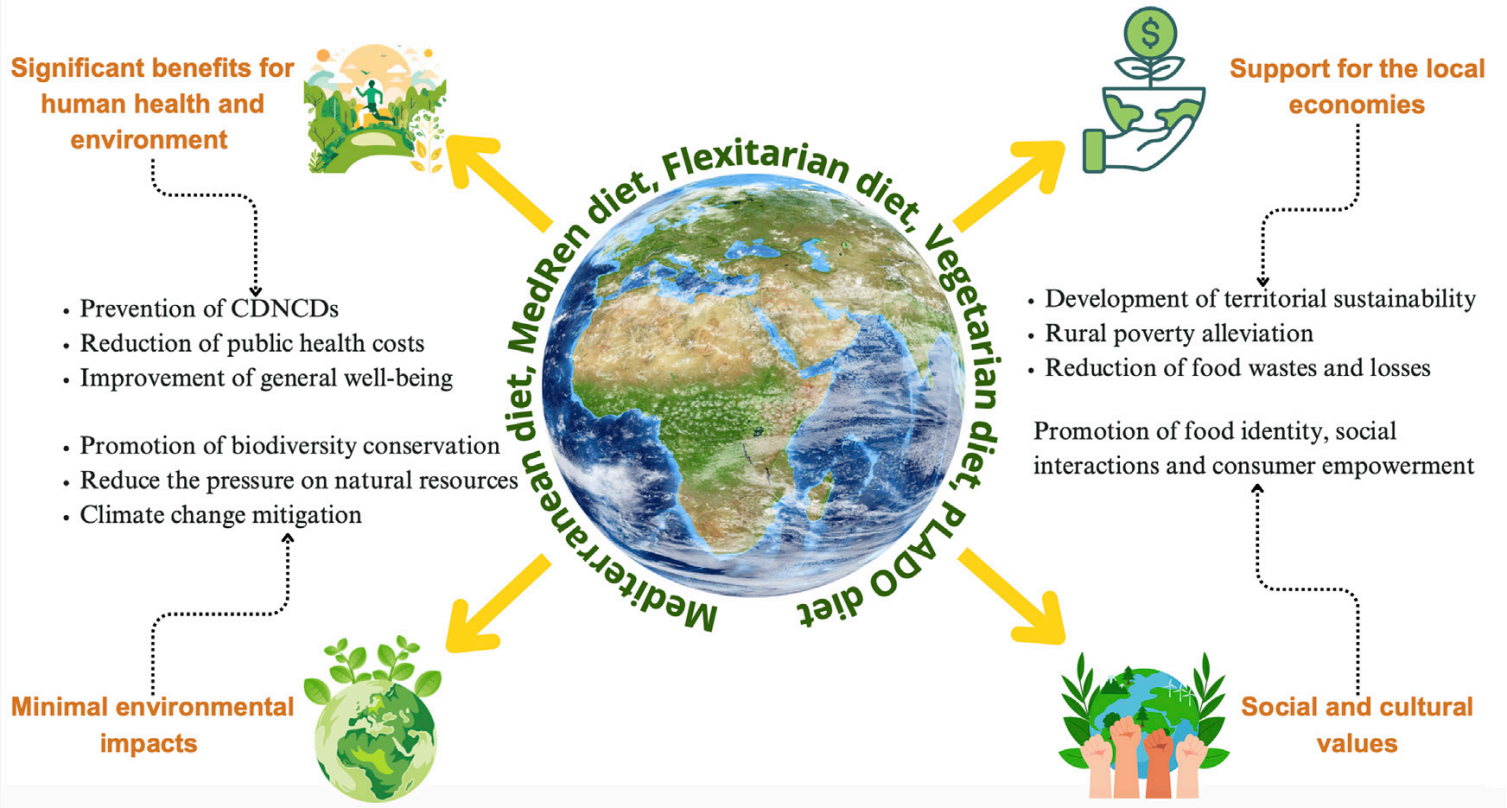
Plant-based diets: key aspects of their sustainability.

Plant protein-based products show also some limitations due to their functional and nutritional properties, to allergens and antinutritional factors and to processing challenges and market limitations (Figure 3).

In order to overcome these limits, it is required a combination of innovative researchers, technological advancements and market diversifications. Refining processing techniques, expanding product ranges and enhancing quality stability will be the pivotal solution for unlocking the full potentiality of the plant protein-based products for food, medical and commodity applications (Fu et al., 2023).

The rising global population has created an urgent demand for economical, abundant, and sustainable foods with the aim to meet nutritional needs. At the same time, the food and agriculture sectors generate massive wastes and by-products at various stages of the supply chain. However, from many of these by-products, with specific and innovative processes, nutrients can be extracted, enhancing their functionality and nutritional values. This approach not only reduces food wastes but also contributes to a sustainable and circular economy by turning wastes into high-value products. Furthermore, it provides a potential strategy for the formulation and prototyping functional foods with healthy beneficial effects, useful in the nutritional treatment of CKD patients (Marrone et al., 2024b; Marrone et al., 2024c). We should also consider that by converting agro-industrial wastes into functional foods with antioxidant and antioxidant capacity, the food industry can reduce its environmental impact, improve food security and create innovative solutions to allow either wastes recycle or the creation of new functional foods, rich in NBCs with healthy benefits (Noce et al., 2021a; Catalfamo et al., 2022; Marrone et al., 2024a; Marrone et al., 2024c; Marrone et al., 2024d).

The concept of environmental sustainability can be applied not only to plant-based proteins but also to plant-based diets.

**FIGURE 4:**
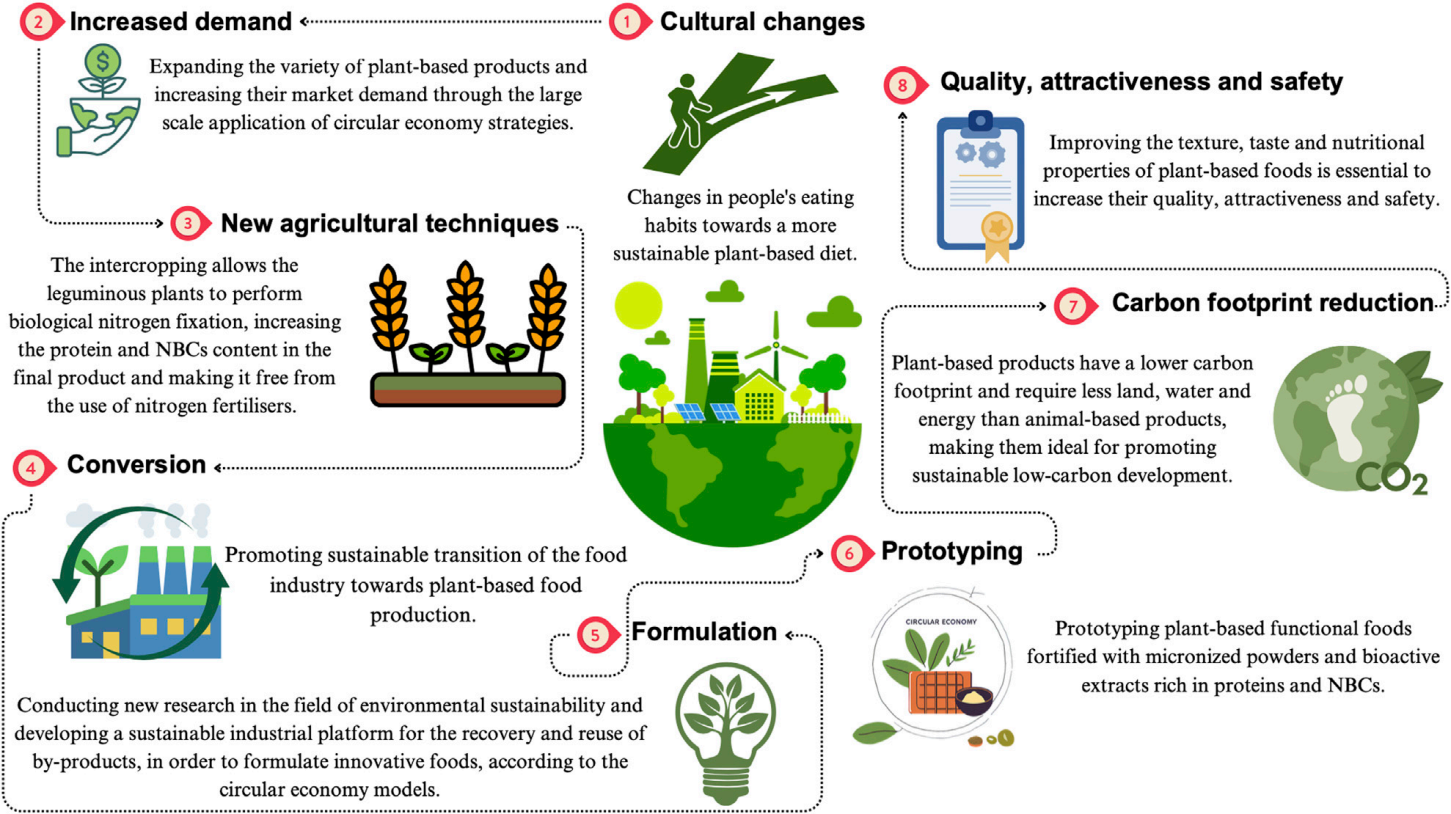
Plant-based products: challenges for the European sustainable food system.

The already discussed dietary patterns, namely, MD, MedRen diet, Flexitarian diet, Vegetarian diet and PLADO diet are recognized as a sustainable food consumption pattern, encompassing several key aspects: i) significant healthy and nutritional benefits, including the prevention of chronic degenerative non-communicable diseases (CDNCDs), reducing public health costs for the CDNCDs management and enhancing the overall wellbeing; ii) minimal environmental impact, promoting the biodiversity conservation, reducing the pressure on natural resources and contributing to the mitigation of climate changes; iii) support for local economies, developing the territorial sustainability, alleviating the rural poverty and reducing the food wastes and losses; iv) social and cultural values, fostering food-related identity, social interactions and consumer empowerment (Figure 4) (Dernini et al., 2017).

**FIGURE 5:**
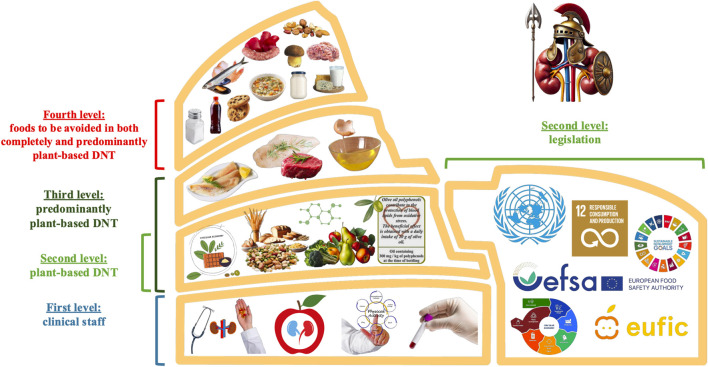
Representation of sustainable nutritional treatment for chronic kidney disease thorough the present Colosseum. As shown in the figure, at the first level of the clinical management of chronic kidney disease patients, there is the clinical staff (nephrologists, nutritionists and/or dietitians, clinical kinesiologists and nurses). The levels two, three and four focus on the dietary nutritional therapy, to define these diets as completely or predominantly plant-based. The second level can be implemented by a legislative part (Food and Agriculture Organization with the implementation of the Agenda 2030 for Sustainable Development Goals, European Food Safety Authority, circular economy models and European Food Information Council), in order to achieve the maximum sustainability from the plant-based dietary nutritional therapy. The combination of the second and third levels outlines a predominantly plant-based dietary nutritional therapy. The fourth level summaries the foods to be avoided in completely or predominantly plant-based dietary nutritional therapy. Abbreviation: DNT, dietary-nutritional therapy.

The already examined diets, if promptly applied and tailored on the clinical characteristics of the patients, represent a useful instrument, combined with the pharmacological therapy, in order to delay the need of RRT (Naber and Purohit, 2021). As shown in Figure 5, the clinical management of CKD patients can be represented by the present Colosseum. The first level is depicted by the clinical staff (nephrologists, nutritionists and/or dietitians, clinical kinesiologists and nurses) required for the treatment, psycho-physical wellbeing and medical care of nephropathic patients. The second, third and fourth levels focus on the DNT. The second plant-based level is represented by fruits and vegetables (with indications on cooking techniques) (Cupisti et al., 2018b), non-whole cereals, legumes, EVOO (containing at least 250 mg of hydroxytyrosol and derivatives *per* kg of product) and foods obtained by circular-economy model. EVOO characterized by high content in minor polar compounds and sustainable and antioxidant functional foods, rich in NBCs, exert innumerable beneficial effects in CKD patients (Noce et al., 2021b; Marrone et al., 2022; Marrone et al., 2024b). This mandatory level can be implemented by a legislation part of European Food Safety Authority, which defines the EVOO health claim able to reduce oxidative stress, to exert antioxidant properties and to protect human body cells and low-density lipoproteins from oxidative damages (EFSA, 2011). Another part of the legislation comes from the European Food Information Council which defines the functional foods and finally, from the 2030 Agenda which sustains “…prevention, reduction, recycling and reuse” (SDG number twelve) of NBCs-rich supply chain by-products useful to formulate functional foods (Romani et al., 2020b). The DNT sustainability reaches its peak if the third level is not considered in the nutritional plan. In fact, a protein-controlled and high-calorie content DNT does not require the implementation of animal-based proteins, as those deriving from red and white meats (with particular attention to those with a medium-high phosphorous content), white fishes and egg whites. The fourth level is mandatory for a proper setting of the DNT, whether it is completely or predominantly plant-based, because it gathers the foods to be avoided.

This is a further strength-point for the plant-based diets sustainability in the clinical management of the CKD patients, as dialysis itself negatively impacts on the environment. At this regard, dialysis leaves a heavy environmental footprint due to water and energy consumption, greenhouse gas emissions and wastes production (Barraclough and McAlister, 2022).

The environmental problems associated with hemodialysis (HD) include high water and energy consumption, CO_2_ emissions, and plastic waste (Vanholder, 2022). Annually, hemodialytic treatments produce around 3.8 tons of CO_2_ *per* patient, primarily from biomedical equipment (37%), energy use (21%), and patient transport (20%). The transport emissions are due to patients’ mobility to reach the dialysis centre from home *and back*, which require vehicles that release CO_2_, CO, NO_x_, and particulate matter (Pelliccia et al., 2011).

HD is a water-intensive treatment that requires purified water to prepare the dialysis fluid of appropriate quality. In the world, for HD, it is assumed that the annual consumption of water is approximately 265 million m^3^. Up to two-thirds of this wastewater consists of the reject water by the reverse osmosis system (176 million m^3^) and by the dialysis machine (Ben Hmida et al., 2023). Water is the main component of dialysate, which establishes the concentration gradient for the diffusive removal of solutes during HD, through filtration steps, ion exchange and reverse osmosis. Additional water is used during the preparation and disinfection cycle of the HD machines (Gauly et al., 2022). In fact, dialysis water treatment should remove chemical and microbial contaminants, since the dialysate effluent is mainly water that contains byproducts of human metabolism (like organic compounds and minerals), possible bacteria, viruses and drugs (Coulliette and Arduino, 2013). The reject water must satisfy all potable water criteria defined by the World Health Organization. In fact, this water is often considered a wastewater, despite being of high quality after the filtering and treatment with activated carbon (Ponson et al., 2014).

Energy consumption is also driven by the facilities’ lighting, cooling and ventilation systems, as well as by heating (using diesel or gas) and air conditioning. The environmental impact also depends on sanitation and laundry services that require water, energy, and cleaning agents (Pelliccia et al., 2011).

Focusing on the huge water consumption in dialysis patients, it is important to reduce the environmental burden for the renal care by implementing virtuous strategies, aimed not only at eco-friendly respect but above all at a healthy lifestyle and an appropriate nutrition.

## 5 Conclusion

CKD is a worldwide spread disease that involves expensive healthcare costs for its management. These costs increase significantly when comorbidities arise or when the disease progresses. A valid strategy to reduce healthcare costs, as well as to increase patients’ survival and to improve their quality of life, is to implement a targeted therapy (pharmacological and non-pharmacological), that aims at slowing the disease progression.

In this context, among the non-pharmacological adjuvant therapies, a key role is played by the nutritional therapy, characterized by a controlled protein intake and by a prevalence of plant-based foods, like MD (in particular the MedRen diet and the Flexitarian diet), the Vegetarian diet and the PLADO diet. Numerous studies have shown that these DNTs exert numerous beneficial properties for CKD patient’s health and for the environmental sustainability.

In fact,

• The NBCs contained in plant-based foods exert antioxidant and anti-inflammatory effects, positively modulate the gut microbiota, improve purine and lipid metabolisms and the body composition of the nephropathic patient. The consumption of plant-based foods in CKD patients is able to reduce drug treatments, to counteract the development of the main complications of the disease and slow down its progression.
• Plant-based foods represent a pivotal strategy for addressing the global challenges of environmental sustainability, human health and food security. Due to their lower environmental impact, superior nutritional profile, and alignment with sustainable practices, plant-based foods offer a valid alternative to traditional animal-based products.
• Innovations in foods processing through the adoption of industrial and sustainable platforms, based on circular economy models, allow the recovery and reuse of supply chain wastes for the formulation and the prototyping of functional foods, rich in NBCs. In this way, food industry can create plant-based functional foods, formulated *ad hoc* for specific patient populations, such as those nephropathic, diabetics, *etc.*, maximising the resource efficiency, reducing the environmental impact and improving food security, concepts that are part of the SDGs of the 2030 Agenda.

In view of what has been stated, the adoption of sustainable food models will allow the containment of the spread of CDNCDs, such as CKD, positively impacting both human health and planet, significantly reducing the costs and resources of the National Health Systems. Moreover, only changes in people’s eating habits towards plant-based diets, by raising awareness among patients, will allow the increasing market demand of plant-based foods and expanding their variety. Therefore, the farmers will be incentivized to adopt new farming techniques that will enable them to create foods rich in NBCs and free of nitrogen fertilizers. At the same time, the existing food companies will be incentivized to convert toward the production of plant-based food. Conducting new research in the fields of nephrology, nutrition and environmental sustainability is the right direction to go, in order to encourage the consumption of a plant-based diet and to formulate *ad hoc* functional foods for specific patient populations, including those with CKD.

## References

[B1] Azizi F. (2018). Tehran lipid and glucose study: a national legacy. Int. J. Endocrinol. Metab. 16 (4 Suppl. l), e84774. 10.5812/ijem.84774 30584440 PMC6289307

[B2] Babich J. S. Dupuis L. Kalantar-Zadeh K. Joshi S. (2023). Hyperkalemia and plant-based diets in chronic kidney disease. Adv. Kidney Dis. Health 30 (6), 487–495. 10.1053/j.akdh.2023.10.001 38453264

[B3] Bach-Faig A. Berry E. M. Lairon D. Reguant J. Trichopoulou A. Dernini S. (2011). Mediterranean diet pyramid today. Science and cultural updates. Public Health Nutr. 14 (12A), 2274–2284. 10.1017/S1368980011002515 22166184

[B4] Bakaloudi D. R. Halloran A. Rippin H. L. Oikonomidou A. C. Dardavesis T. I. Williams J. (2021). Intake and adequacy of the vegan diet. A systematic review of the evidence. Clin. Nutr. 40 (5), 3503–3521. 10.1016/j.clnu.2020.11.035 33341313

[B5] Barraclough K. A. McAlister S. (2022). Assessing the carbon footprint of hemodialysis: a first step toward environmentally sustainable kidney care. J. Am. Soc. Nephrol. 33 (9), 1635–1637. 10.1681/ASN.2022060661 35840174 PMC9529175

[B6] Barsotti G. Morelli E. Cupisti A. Meola M. Dani L. Giovannetti S. (1996). A low-nitrogen low-phosphorus Vegan diet for patients with chronic renal failure. Nephron 74 (2), 390–394. 10.1159/000189341 8893161

[B7] Ben Hmida M. Mechichi T. Piccoli G. B. Ksibi M. (2023). Water implications in dialysis therapy, threats and opportunities to reduce water consumption: a call for the planet. Kidney Int. 104 (1), 46–52. 10.1016/j.kint.2023.04.008 37116701

[B8] Blackburn H. (2017). Invited commentary: 30-year perspective on the seven countries study. Am. J. Epidemiol. 185 (11), 1143–1147. 10.1093/aje/kwx071 28535176

[B9] Blaha M. J. DeFilippis A. P. (2021). Multi-ethnic study of atherosclerosis (MESA): JACC focus seminar 5/8. J. Am. Coll. Cardiol. 77 (25), 3195–3216. 10.1016/j.jacc.2021.05.006 34167645 PMC8091185

[B10] Brenner B. M. Lawler E. V. Mackenzie H. S. (1996). The hyperfiltration theory: a paradigm shift in nephrology. Kidney Int. 49 (6), 1774–1777. 10.1038/ki.1996.265 8743495

[B11] Bruns A. Greupner T. Nebl J. Hahn A. (2024). Plant-based diets and cardiovascular risk factors: a comparison of flexitarians, vegans and omnivores in a cross-sectional study. BMC Nutr. 10 (1), 29. 10.1186/s40795-024-00839-9 38347653 PMC10860304

[B12] Burlingame B. Dernini S. Charrondiere U. Stadlmayr B. Mondovi S. Dop M. (2011). Biodiversity and sustainable diets. Rome, Italy: Food and Agriculture Organization of the United Nations.

[B13] Cannata-Andia J. B. Martin-Carro B. Martin-Virgala J. Rodriguez-Carrio J. Bande-Fernandez J. J. Alonso-Montes C. (2021). Chronic kidney disease-mineral and bone disorders: pathogenesis and management. Calcif. Tissue Int. 108 (4), 410–422. 10.1007/s00223-020-00777-1 33190187

[B14] CarbonTrust (2021). Quorn footprint comparison report. Available online at: https://www.quorn.co.uk/assets/files/content/Carbon-Trust-Comparison-Report-2021.pdf (Accessed January 14, 2025).

[B15] Catalfamo L. Marrone G. Basilicata M. Vivarini I. Paolino V. Della-Morte D. (2022). The utility of capsicum annuum L. in internal medicine and in dentistry: a comprehensive review. Int. J. Environ. Res. Public Health 19, 11187. 10.3390/ijerph191811187 36141454 PMC9517535

[B16] Chadban S. Arici M. Power A. Wu M. S. Mennini F. S. Arango Alvarez J. J. (2024). Projecting the economic burden of chronic kidney disease at the patient level (Inside CKD): a microsimulation modelling study. EClinicalMedicine 72, 102615. 10.1016/j.eclinm.2024.102615 39010976 PMC11247148

[B17] Chang L. L. Rhee C. M. Kalantar-Zadeh K. Woodrow G. (2024). Dietary protein restriction in patients with chronic kidney disease. N. Engl. J. Med. 390 (1), 86–89. 10.1056/NEJMclde2304134 38169496

[B18] Chauveau P. Aparicio M. Bellizzi V. Campbell K. Hong X. Johansson L. (2018). Mediterranean diet as the diet of choice for patients with chronic kidney disease. Nephrol. Dial. Transpl. 33 (5), 725–735. 10.1093/ndt/gfx085 29106612

[B19] Chauveau P. Koppe L. Combe C. Lasseur C. Trolonge S. Aparicio M. (2019). Vegetarian diets and chronic kidney disease. Nephrol. Dial. Transpl. 34 (2), 199–207. 10.1093/ndt/gfy164 29982610

[B20] Cho K. S. Ko I. K. Yoo J. J. (2018). Bioactive compounds for the treatment of renal disease. Yonsei Med. J. 59 (9), 1015–1025. 10.3349/ymj.2018.59.9.1015 30328315 PMC6192891

[B21] Cicero A. F. Nascetti S. Lopez-Sabater M. C. Elosua R. Salonen J. T. Nyyssonen K. (2008). Changes in LDL fatty acid composition as a response to olive oil treatment are inversely related to lipid oxidative damage: the EUROLIVE study. J. Am. Coll. Nutr. 27 (2), 314–320. 10.1080/07315724.2008.10719705 18689564

[B22] Cigarran Guldris S. Latorre Catala J. A. Sanjurjo Amado A. Menendez Granados N. Pineiro Varela E. (2022). Fibre intake in chronic kidney disease: what fibre should we recommend? Nutrients 14 (20), 4419. 10.3390/nu14204419 36297103 PMC9612304

[B23] Coulliette A. D. Arduino M. J. (2013). Hemodialysis and water quality. Semin. Dial. 26 (4), 427–438. 10.1111/sdi.12113 23859187 PMC4596525

[B24] Cupisti A. Bottai A. Bellizzi V. Brunori G. Cianciaruso B. De Nicola L. (2015). Characteristics of patients with chronic kidney disease referred to a nephrology outpatient clinic: results of Nefrodata study. G. Ital. Nefrol. 32 (2), gin/32.2.36. 26005945

[B25] Cupisti A. Brunori G. Di Iorio B. R. D'Alessandro C. Pasticci F. Cosola C. (2018a). Nutritional treatment of advanced CKD: twenty consensus statements. J. Nephrol. 31 (4), 457–473. 10.1007/s40620-018-0497-z 29797247 PMC6061255

[B26] Cupisti A. Giannese D. Moriconi D. D'Alessandro C. Torreggiani M. Piccoli G. B. (2020). Nephroprotection by SGLT2i in CKD patients: may it be modulated by low-protein plant-based diets? Front. Med. (Lausanne) 7, 622593. 10.3389/fmed.2020.622593 33425967 PMC7793896

[B27] Cupisti A. Kalantar-Zadeh K. (2013). Management of natural and added dietary phosphorus burden in kidney disease. Semin. Nephrol. 33 (2), 180–190. 10.1016/j.semnephrol.2012.12.018 23465504 PMC5797670

[B28] Cupisti A. Kovesdy C. P. D'Alessandro C. Kalantar-Zadeh K. (2018b). Dietary approach to recurrent or chronic hyperkalaemia in patients with decreased kidney function. Nutrients 10 (3), 261. 10.3390/nu10030261 29495340 PMC5872679

[B29] Dabek B. Dybiec J. Frak W. Fularski P. Lisinska W. Radzioch E. (2023). Novel therapeutic approaches in the management of chronic kidney disease. Biomedicines 11 (10), 2746. 10.3390/biomedicines11102746 37893119 PMC10604464

[B30] D'Alessandro C. Giannese D. Panichi V. Cupisti A. (2023). Mediterranean dietary pattern adjusted for CKD patients: the MedRen diet. Nutrients 15 (5), 1256. 10.3390/nu15051256 36904256 PMC10005115

[B31] De Angelis S. Noce A. Di Renzo L. Cianci R. Naticchia A. Giarrizzo G. F. (2007). Is rasburicase an effective alternative to allopurinol for management of hyperuricemia in renal failure patients? A double blind-randomized study. Eur. Rev. Med. Pharmacol. Sci. 11 (3), 179–184. 17970234

[B32] De Bhailis A. M. Kalra P. A. (2022). Hypertension and the kidneys. Br. J. Hosp. Med. (Lond) 83 (5), 1–11. 10.12968/hmed.2021.0440 35653320

[B33] De Lorenzo A. Noce A. Bigioni M. Calabrese V. Della Rocca D. G. Di Daniele N. (2010). The effects of Italian Mediterranean organic diet (IMOD) on health status. Curr. Pharm. Des. 16 (7), 814–824. 10.2174/138161210790883561 20388092

[B34] Demirci B. G. Tutal E. Eminsoy I. O. Kulah E. Sezer S. (2019). Dietary fiber intake: its relation with glycation end products and arterial stiffness in end-stage renal disease patients. J. Ren. Nutr. 29 (2), 136–142. 10.1053/j.jrn.2018.08.007 30314838

[B35] Dernini S. Berry E. M. Serra-Majem L. La Vecchia C. Capone R. Medina F. X. (2017). Med Diet 4.0: the Mediterranean diet with four sustainable benefits. Public Health Nutr. 20 (7), 1322–1330. 10.1017/S1368980016003177 28003037 PMC10261651

[B36] Dessi M. Noce A. Bertucci P. Noce G. Rizza S. De Stefano A. (2014). Plasma and erythrocyte membrane phospholipids and fatty acids in Italian general population and hemodialysis patients. Lipids Health Dis. 13, 54. 10.1186/1476-511X-13-54 24655786 PMC4234015

[B37] Dinu M. Abbate R. Gensini G. F. Casini A. Sofi F. (2017). Vegetarian, vegan diets and multiple health outcomes: a systematic review with meta-analysis of observational studies. Crit. Rev. Food Sci. Nutr. 57 (17), 3640–3649. 10.1080/10408398.2016.1138447 26853923

[B38] EAT (2025). “EAT-lancet commission summary report,” in P. The EAT-lancet commission on food, health. Oslo, Norway: EAT.

[B39] EFSA (2011). Scientific Opinion on the substantiation of health claims related to olive oil and maintenance of normal blood LDL-cholesterol concentrations (ID 1316, 1332), maintenance of normal (fasting) blood concentrations of triglycerides (ID 1316, 1332), maintenance of normal blood HDL cholesterol concentrations (ID 1316, 1332) and maintenance of normal blood glucose concentrations (ID 4244) pursuant to Article 13(1) of Regulation (EC) No 1924/2006. Available online at: https://www.efsa.europa.eu/en/efsajournal/pub/2044 (Accessed January 14, 2025). 10.2903/j.efsa.2011.2044 PMC13129925 42078784

[B40] European Commission (2022). Commission implementing decision of 9.11.2022, on the selection of simple programmes for the promotion of agricultural products for 2022 under regulation (EU) No 1144/2014 of the European Parliament and of the Council. Brussels: European Commission.

[B41] FAO (2019). Climate change and the global dairy cattle sector. Rome, Italy: FAO.

[B42] FAO (2023). Daily protein supply from animal and plant-based foods, European Union (27), 1961 to 2021. Available online at: https://ourworldindata.org/grapher/daily-protein-supply-from-animal-and-plant-based-foods?country=∼OWID_EU27 (Accessed March 1, 2025).

[B43] Fouque D. Laville M. (2009). Low protein diets for chronic kidney disease in non diabetic adults. Cochrane Database Syst. Rev. 19 (3), CD001892. 10.1002/14651858.CD001892.pub3 19588328

[B44] Fu Q. Zhao J. Rong S. Han Y. Liu F. Chu Q. (2023). Research advances in plant protein-based products: protein sources, processing technology, and food applications. J. Agric. Food Chem. 71 (42), 15429–15444. 10.1021/acs.jafc.3c02224 37824166

[B45] Garibotto G. Sofia A. Parodi E. L. Ansaldo F. Bonanni A. Picciotto D. (2018). Effects of low-protein, and supplemented very low-protein diets, on muscle protein turnover in patients with CKD. Kidney Int. Rep. 3 (3), 701–710. 10.1016/j.ekir.2018.01.003 29854979 PMC5976852

[B46] Gauly A. Fleck N. Kircelli F. (2022). Advanced hemodialysis equipment for more eco-friendly dialysis. Int. Urol. Nephrol. 54 (5), 1059–1065. 10.1007/s11255-021-02981-w 34480255 PMC9005388

[B47] George C. Mogueo A. Okpechi I. Echouffo-Tcheugui J. B. Kengne A. P. (2017). Chronic kidney disease in low-income to middle-income countries: the case for increased screening. BMJ Glob. Health 2 (2), e000256. 10.1136/bmjgh-2016-000256 PMC5584488 29081996

[B48] Giannese D. D'Alessandro C. Panichi V. Pellegrino N. Cupisti A. (2023). Nutritional treatment as a synergic intervention to pharmacological therapy in CKD patients. Nutrients 15 (12), 2715. 10.3390/nu15122715 37375619 PMC10302813

[B49] Giovannetti S. Maggiore Q. (1964). A low-nitrogen diet with proteins of high biological value for severe chronic uraemia. Lancet 1 (7341), 1000–1003. 10.1016/s0140-6736(64)91919-1 14129799

[B50] Gluba-Brzozka A. Franczyk B. Rysz J. (2017). Vegetarian diet in chronic kidney disease-A friend or foe. Nutrients 9 (4), 374. 10.3390/nu9040374 28394274 PMC5409713

[B51] Golestaneh L. Alvarez P. J. Reaven N. L. Funk S. E. McGaughey K. J. Romero A. (2017). All-cause costs increase exponentially with increased chronic kidney disease stage. Am. J. Manag. Care 23 (10 Suppl. l), S163–S172. 28978205

[B52] Grazioli E. Romani A. Marrone G. Di Lauro M. Cerulli C. Urciuoli S. (2021). Impact of physical activity and natural bioactive compounds on endothelial dysfunction in chronic kidney disease. Life (Basel) 11 (8), 841. 10.3390/life11080841 34440585 PMC8402113

[B53] Grazioli E. Tranchita E. Marrone G. Urciuoli S. Di Lauro M. Cerulli C. (2022). The impact of functional bars and adapted physical activity on quality of life in chronic kidney disease: a pilot study. Int. J. Environ. Res. Public Health 19 (6), 3281. 10.3390/ijerph19063281 35328973 PMC8953183

[B54] Gupta A. Nagaraju S. P. Bhojaraja M. V. Swaminathan S. M. Mohan P. B. (2023). Hypertension in chronic kidney disease: an update on diagnosis and management. South Med. J. 116 (2), 237–244. 10.14423/SMJ.0000000000001516 36724542

[B55] Haghighatdoost F. Bellissimo N. Totosy de Zepetnek J. O. Rouhani M. H. (2017). Association of vegetarian diet with inflammatory biomarkers: a systematic review and meta-analysis of observational studies. Public Health Nutr. 20 (15), 2713–2721. 10.1017/S1368980017001768 28836492 PMC10261540

[B56] Haring B. Selvin E. Liang M. Coresh J. Grams M. E. Petruski-Ivleva N. (2017). Dietary protein sources and risk for incident chronic kidney disease: results from the atherosclerosis risk in communities (ARIC) study. J. Ren. Nutr. 27 (4), 233–242. 10.1053/j.jrn.2016.11.004 28065493 PMC5476496

[B57] Heerspink H. J. L. Stefansson B. V. Correa-Rotter R. Chertow G. M. Greene T. Hou F. F. (2020). Dapagliflozin in patients with chronic kidney disease. N. Engl. J. Med. 383 (15), 1436–1446. 10.1056/NEJMoa2024816 32970396

[B58] Hertzler S. R. Lieblein-Boff J. C. Weiler M. Allgeier C. (2020). Plant proteins: assessing their nutritional quality and effects on health and physical function. Nutrients 12 (12), 3704. 10.3390/nu12123704 33266120 PMC7760812

[B59] Hounkpatin H. O. Harris S. Fraser S. D. S. Day J. Mindell J. S. Taal M. W. (2020). Prevalence of chronic kidney disease in adults in England: comparison of nationally representative cross-sectional surveys from 2003 to 2016. BMJ Open 10 (8), e038423. 10.1136/bmjopen-2020-038423 PMC7430464 32792448

[B60] Ikizler T. A. Burrowes J. D. Byham-Gray L. D. Campbell K. L. Carrero J. J. Chan W. (2020). KDOQI clinical practice guideline for nutrition in CKD: 2020 update. Am. J. Kidney Dis. 76 (3 Suppl. 1), S1–S107. 10.1053/j.ajkd.2020.05.006 32829751

[B61] Jacobs C. (2009). Renal replacement therapy by hemodialysis: an overview. Nephrol. Ther. 5 (4), 306–312. 10.1016/j.nephro.2009.03.001 19481513

[B62] Jaeger S. R. Cardello A. V. Jin D. Ryan G. S. Giacalone D. (2023). Consumer perception of plant-based yoghurt: sensory drivers of liking and emotional, holistic and conceptual associations. Food Res. Int. 167, 112666. 10.1016/j.foodres.2023.112666 37087252

[B63] Jager K. J. Kovesdy C. Langham R. Rosenberg M. Jha V. Zoccali C. (2019). A single number for advocacy and communication-worldwide more than 850 million individuals have kidney diseases. Kidney Int. 96 (5), 1048–1050. 10.1016/j.kint.2019.07.012 31582227

[B64] Jankowski J. Floege J. Fliser D. Bohm M. Marx N. (2021). Cardiovascular disease in chronic kidney disease: pathophysiological insights and therapeutic options. Circulation 143 (11), 1157–1172. 10.1161/CIRCULATIONAHA.120.050686 33720773 PMC7969169

[B65] Jha V. Al-Ghamdi S. M. G. Li G. Wu M. S. Stafylas P. Retat L. (2023). Global economic burden associated with chronic kidney disease: a pragmatic review of medical costs for the inside CKD research programme. Adv. Ther. 40 (10), 4405–4420. 10.1007/s12325-023-02608-9 37493856 PMC10499937

[B66] Jimenez-Lopez C. Carpena M. Lourenco-Lopes C. Gallardo-Gomez M. Lorenzo J. M. Barba F. J. (2020). Bioactive compounds and quality of extra virgin olive oil. Foods 9 (8), 1014. 10.3390/foods9081014 32731481 PMC7466243

[B67] Jommi C. Armeni P. Battista M. di Procolo P. Conte G. Ronco C. (2018). The cost of patients with chronic kidney failure before dialysis: results from the IRIDE observational study. Pharmacoecon Open 2 (4), 459–467. 10.1007/s41669-017-0062-z 29623638 PMC6249198

[B68] Joshi S. Hashmi S. Shah S. Kalantar-Zadeh K. (2020). Plant-based diets for prevention and management of chronic kidney disease. Curr. Opin. Nephrol. Hypertens. 29 (1), 16–21. 10.1097/MNH.0000000000000574 31725014

[B69] Joshi S. McMacken M. Kalantar-Zadeh K. (2021). Plant-based diets for kidney disease: a guide for clinicians. Am. J. Kidney Dis. 77 (2), 287–296. 10.1053/j.ajkd.2020.10.003 33075387

[B70] Kalantar-Zadeh K. Fouque D. (2017). Nutritional management of chronic kidney disease. N. Engl. J. Med. 377 (18), 1765–1776. 10.1056/NEJMra1700312 29091561

[B71] Kalantar-Zadeh K. Joshi S. Schlueter R. Cooke J. Brown-Tortorici A. Donnelly M. (2020). Plant-dominant low-protein diet for conservative management of chronic kidney disease. Nutrients 12 (7), 1931. 10.3390/nu12071931 32610641 PMC7400005

[B72] Kalantar-Zadeh K. Rhee C. M. Joshi S. Brown-Tortorici A. Kramer H. M. (2022). Medical nutrition therapy using plant-focused low-protein meal plans for management of chronic kidney disease in diabetes. Curr. Opin. Nephrol. Hypertens. 31 (1), 26–35. 10.1097/MNH.0000000000000761 34750331

[B73] Kasiske B. L. Lakatua J. D. Ma J. Z. Louis T. A. (1998). A meta-analysis of the effects of dietary protein restriction on the rate of decline in renal function. Am. J. Kidney Dis. 31 (6), 954–961. 10.1053/ajkd.1998.v31.pm9631839 9631839

[B74] Katz D. L. Meller S. (2014). Can we say what diet is best for health? Annu. Rev. Public Health 35, 83–103. 10.1146/annurev-publhealth-032013-182351 24641555

[B75] Key T. J. Papier K. Tong T. Y. N. (2022). Plant-based diets and long-term health: findings from the EPIC-Oxford study. Proc. Nutr. Soc. 81 (2), 190–198. 10.1017/S0029665121003748 35934687 PMC7613518

[B76] Keys A. Menotti A. Aravanis C. Blackburn H. Djordevic B. S. Buzina R. (1984). The seven countries study: 2,289 deaths in 15 years. Prev. Med. 13 (2), 141–154. 10.1016/0091-7435(84)90047-1 6739443

[B77] Kidney Disease: Improving Global Outcomes (KDIGO) CKD Work Group (2024). KDIGO 2024 clinical practice guideline for the evaluation and management of chronic kidney disease. Kidney Int. 105 (4S), S117–S314. 10.1016/j.kint.2023.10.018 38490803

[B78] Klahr S. Levey A. S. Beck G. J. Caggiula A. W. Hunsicker L. Kusek J. W. (1994). The effects of dietary protein restriction and blood-pressure control on the progression of chronic renal disease. Modification of Diet in Renal Disease Study Group. N. Engl. J. Med. 330 (13), 877–884. 10.1056/NEJM199403313301301 8114857

[B79] Ko G. J. Kalantar-Zadeh K. (2021). How important is dietary management in chronic kidney disease progression? A role for low protein diets. Korean J. Intern Med. 36 (4), 795–806. 10.3904/kjim.2021.197 34153180 PMC8273814

[B80] Koppe L. Fouque D. (2019). The role for protein restriction in addition to renin-angiotensin-aldosterone system inhibitors in the management of CKD. Am. J. Kidney Dis. 73 (2), 248–257. 10.1053/j.ajkd.2018.06.016 30149957

[B81] Koppe L. Soulage C. O. (2022). The impact of dietary nutrient intake on gut microbiota in the progression and complications of chronic kidney disease. Kidney Int. 102 (4), 728–739. 10.1016/j.kint.2022.06.025 35870642

[B82] Kovesdy C. P. (2022). Epidemiology of chronic kidney disease: an update 2022. Kidney Int. 12 (1), 7–11. 10.1016/j.kisu.2021.11.003 PMC9073222 35529086

[B83] Kraak V. I. Aschemann-Witzel J. (2024). The future of plant-based diets: aligning healthy marketplace choices with equitable, resilient, and sustainable food systems. Annu. Rev. Public Health 45 (1), 253–275. 10.1146/annurev-publhealth-060722-032021 38772624

[B84] Kumar M. Dev S. Khalid M. U. Siddenthi S. M. Noman M. John C. (2023). The bidirectional link between diabetes and kidney disease: mechanisms and management. Cureus 15 (9), e45615. 10.7759/cureus.45615 37868469 PMC10588295

[B85] Lamb E. J. Levey A. S. Stevens P. E. (2013). The Kidney Disease Improving Global Outcomes (KDIGO) guideline update for chronic kidney disease: evolution not revolution. Clin. Chem. 59 (3), 462–465. 10.1373/clinchem.2012.184259 23449698

[B86] Lane K. E. Wilson M. Hellon T. G. Davies I. G. (2022). Bioavailability and conversion of plant based sources of omega-3 fatty acids - a scoping review to update supplementation options for vegetarians and vegans. Crit. Rev. Food Sci. Nutr. 62 (18), 4982–4997. 10.1080/10408398.2021.1880364 33576691

[B87] Lau W. L. Kalantar-Zadeh K. Vaziri N. D. (2015). The gut as a source of inflammation in chronic kidney disease. Nephron 130 (2), 92–98. 10.1159/000381990 25967288 PMC4485546

[B88] Lau W. L. Savoj J. Nakata M. B. Vaziri N. D. (2018). Altered microbiome in chronic kidney disease: systemic effects of gut-derived uremic toxins. Clin. Sci. (Lond) 132 (5), 509–522. 10.1042/CS20171107 29523750

[B89] Li Y. Barve K. Cockrell M. Agarwal A. Casebeer A. Dixon S. W. (2023). Managing comorbidities in chronic kidney disease reduces utilization and costs. BMC Health Serv. Res. 23 (1), 1418. 10.1186/s12913-023-10424-8 38102650 PMC10722800

[B90] Lv J. C. Zhang L. X. (2019). Prevalence and disease burden of chronic kidney disease. Adv. Exp. Med. Biol. 1165, 3–15. 10.1007/978-981-13-8871-2_1 31399958

[B91] Marrone G. Basilicata M. Lauro M. Vita C. Masci C. Klinger F. (2024a). Healthy effects of pomegranate (punica granatum L.) in internal medicine and dentistry. Appl. Sci. (Basel). 14, 1570. 10.3390/app14041570

[B92] Marrone G. Guerriero C. Palazzetti D. Lido P. Marolla A. Di Daniele F. (2021). Vegan diet health benefits in metabolic syndrome. Nutrients 13 (3), 817. 10.3390/nu13030817 33801269 PMC7999488

[B93] Marrone G. Murri A. Urciuoli S. Di Lauro M. Grazioli E. Vignolini P. (2024b). Functional foods and adapted physical activity as new adjuvant therapy for chronic kidney disease patients. Nutrients 16 (14), 2325. 10.3390/nu16142325 39064768 PMC11279472

[B94] Marrone G. Urciuoli S. Di Lauro M. Cornali K. Masci C. Tesauro M. (2024c). The possible role of plant-based bars consumption in CKD geriatric patients. Pharm. (Basel) 17 (12), 1689. 10.3390/ph17121689 PMC11677204 39770530

[B95] Marrone G. Urciuoli S. Di Lauro M. Cornali K. Montalto G. Masci C. (2024d). Saffron (*Crocus sativus* L.) and its by-products: healthy effects in internal medicine. Nutrients 16 (14), 2319. 10.3390/nu16142319 39064764 PMC11279474

[B96] Marrone G. Urciuoli S. Di Lauro M. Ruzzolini J. Ieri F. Vignolini P. (2022). Extra virgin olive oil and cardiovascular protection in chronic kidney disease. Nutrients 14 (20), 4265. 10.3390/nu14204265 36296948 PMC9607338

[B97] Martinez-Gonzalez M. A. Salas-Salvado J. Estruch R. Corella D. Fito M. Ros E. (2015). Benefits of the mediterranean diet: insights from the PREDIMED study. Prog. Cardiovasc Dis. 58 (1), 50–60. 10.1016/j.pcad.2015.04.003 25940230

[B98] Medina-Vera I. Avila-Nava A. Leon-Lopez L. Gutierrez-Solis A. L. Talamantes-Gomez J. M. Marquez-Mota C. C. (2024). Plant-based proteins: clinical and technological importance. Food Sci. Biotechnol. 33 (11), 2461–2475. 10.1007/s10068-024-01600-5 39144188 PMC11319542

[B99] Meena V. S. Meena S. K. Verma J. P. Kumar A. Aeron A. Mishra P. K. (2017). Plant beneficial rhizospheric microorganism (PBRM) strategies to improve nutrients use efficiency: a review. Ecol. Eng. 107, 8–32. 10.1016/j.ecoleng.2017.06.058

[B100] Mennini F. S. Russo S. Marcellusi A. Quintaliani G. Fouque D. (2014). Economic effects of treatment of chronic kidney disease with low-protein diet. J. Ren. Nutr. 24 (5), 313–321. 10.1053/j.jrn.2014.05.003 25167997

[B101] Menotti A. Puddu P. E. (2015). How the Seven Countries Study contributed to the definition and development of the Mediterranean diet concept: a 50-year journey. Nutr. Metab. Cardiovasc Dis. 25 (3), 245–252. 10.1016/j.numecd.2014.12.001 25650160

[B102] Mills K. T. Xu Y. Zhang W. Bundy J. D. Chen C. S. Kelly T. N. (2015). A systematic analysis of worldwide population-based data on the global burden of chronic kidney disease in 2010. Kidney Int. 88 (5), 950–957. 10.1038/ki.2015.230 26221752 PMC4653075

[B103] Ministero della Salute (2020). Malattia renale cronica. Available online at: https://www.salute.gov.it/portale/news/p3_2_3_1_1.jsp?lingua=italiano&menu=dossier&p=dadossier&id=65#:∼:text=In%20Italia%20la%20prevalenza%20nella,3%25%20(fig%201) (Accessed February 26, 2025).

[B104] Naber T. Purohit S. (2021). Chronic kidney disease: role of diet for a reduction in the severity of the disease. Nutrients 13 (9), 3277. 10.3390/nu13093277 34579153 PMC8467342

[B105] Narasaki Y. Kalantar-Zadeh K. Rhee C. M. Brunori G. Zarantonello D. (2023). Vegetarian nutrition in chronic kidney disease. Nutrients 16 (1), 66. 10.3390/nu16010066 38201898 PMC10780746

[B106] Narasaki Y. Rhee C. M. (2020). Dietary therapy for managing hyperphosphatemia. Clin. J. Am. Soc. Nephrol. 16 (1), 9–11. 10.2215/CJN.18171120 33380472 PMC7792640

[B107] Natesan V. Kim S. J. (2025). Natural compounds in kidney disease: therapeutic potential and drug development. Biomol. Ther. Seoul. 33 (1), 39–53. 10.4062/biomolther.2024.142 39632648 PMC11704401

[B108] Nawaz S. Chinnadurai R. Al-Chalabi S. Evans P. Kalra P. A. Syed A. A. (2023). Obesity and chronic kidney disease: a current review. Obes. Sci. Pract. 9 (2), 61–74. 10.1002/osp4.629 37034567 PMC10073820

[B109] Nelson M. E. Hamm M. W. Hu F. B. Abrams S. A. Griffin T. S. (2016). Alignment of healthy dietary patterns and environmental sustainability: a systematic review. Adv. Nutr. 7 (6), 1005–1025. 10.3945/an.116.012567 28140320 PMC5105037

[B110] Noce A. Di Daniele F. Campo M. Di Lauro M. Pietroboni Zaitseva A. Di Daniele N. (2021a). Effect of hydrolysable tannins and anthocyanins on recurrent urinary tract infections in nephropathic patients: preliminary data. Nutrients 13 (2), 591. 10.3390/nu13020591 33670236 PMC7916964

[B111] Noce A. Marchetti M. Marrone G. Di Renzo L. Di Lauro M. Di Daniele F. (2022). Link between gut microbiota dysbiosis and chronic kidney disease. Eur. Rev. Med. Pharmacol. Sci. 26 (6), 2057–2074. 10.26355/eurrev_202203_28354 35363356

[B112] Noce A. Marrone G. Urciuoli S. Di Daniele F. Di Lauro M. Pietroboni Zaitseva A. (2021b). Usefulness of extra virgin olive oil minor polar compounds in the management of chronic kidney disease patients. Nutrients 13 (2), 581. 10.3390/nu13020581 33578682 PMC7916323

[B113] Noce A. Marrone G. Wilson Jones G. Di Lauro M. Pietroboni Zaitseva A. Ramadori L. (2021c). Nutritional approaches for the management of metabolic acidosis in chronic kidney disease. Nutrients 13 (8), 2534. 10.3390/nu13082534 34444694 PMC8401674

[B114] Palli D. Berrino F. Vineis P. Tumino R. Panico S. Masala G. (2003). A molecular epidemiology project on diet and cancer: the EPIC-Italy Prospective Study. Design and baseline characteristics of participants. Tumori 89 (6), 586–593. 10.1177/030089160308900602 14870823

[B115] Patel K. P. Luo F. J. Plummer N. S. Hostetter T. H. Meyer T. W. (2012). The production of p-cresol sulfate and indoxyl sulfate in vegetarians versus omnivores. Clin. J. Am. Soc. Nephrol. 7 (6), 982–988. 10.2215/CJN.12491211 22490877 PMC3362314

[B116] Pelliccia F. Parisotto M.-T. Miriunis C. (2011). Environmental guidelines for dialysis: a practical guide to reduce the environmental burden of dialysis.

[B117] Pellinen T. Paivarinta E. Isotalo J. Lehtovirta M. Itkonen S. T. Korkalo L. (2022). Replacing dietary animal-source proteins with plant-source proteins changes dietary intake and status of vitamins and minerals in healthy adults: a 12-week randomized controlled trial. Eur. J. Nutr. 61 (3), 1391–1404. 10.1007/s00394-021-02729-3 34837522 PMC8921037

[B118] Perez-Torres A. Caverni-Munoz A. Gonzalez Garcia E. (2022). Mediterranean diet and chronic kidney disease (CKD): a practical approach. Nutrients 15 (1), 97. 10.3390/nu15010097 36615755 PMC9824533

[B119] Pett K. D. Willett W. C. Vartiainen E. Katz D. L. (2017). The seven countries study. Eur. Heart J. 38 (42), 3119–3121. 10.1093/eurheartj/ehx603 29121230

[B120] Pighin D. Pazos A. Chamorro V. Paschetta F. Cunzolo S. Godoy F. (2016). A contribution of beef to human health: a review of the role of the animal production systems. ScientificWorldJournal 2016, 8681491. 10.1155/2016/8681491 26989765 PMC4771914

[B121] Pogorzelska-Nowicka E. Atanasov A. G. Horbanczuk J. Wierzbicka A. (2018). Bioactive compounds in functional meat products. Molecules 23 (2), 307. 10.3390/molecules23020307 29385097 PMC6017222

[B122] Ponson L. Arkouche W. Laville M. (2014). Toward green dialysis: focus on water savings. Hemodial. Int. 18 (1), 7–14. 10.1111/hdi.12117 24319997

[B123] Poore J. Nemecek T. (2018). Reducing food's environmental impacts through producers and consumers. Science 360 (6392), 987–992. 10.1126/science.aaq0216 29853680

[B124] Riboli E. Kaaks R. (1997). The EPIC project: rationale and study design. European prospective investigation into cancer and nutrition. Int. J. Epidemiol. 26 (Suppl. 1), S6–S14. 10.1093/ije/26.suppl_1.s6 9126529

[B125] Rodrigues Neto Angeloco L. Arces de Souza G. C. Almeida Romao E. Garcia Chiarello P. (2018). Alkaline diet and metabolic acidosis: practical approaches to the nutritional management of chronic kidney disease. J. Ren. Nutr. 28 (3), 215–220. 10.1053/j.jrn.2017.10.006 29221627

[B126] Romani A. Bernini R. Noce A. Urciuoli S. Di Lauro M. Pietroboni Zaitseva A. (2020a). Potential beneficial effects of extra virgin olive oils characterized by high content in minor polar compounds in nephropathic patients: a pilot study. Molecules 25 (20), 4757. 10.3390/molecules25204757 33081292 PMC7587576

[B127] Romani A. Campo M. Urciuoli S. Marrone G. Noce A. Bernini R. (2020b). An industrial and sustainable platform for the production of bioactive micronized powders and extracts enriched in polyphenols from Olea europaea L. and Vitis vinifera L. Wastes. Front. Nutr. 7, 120. 10.3389/fnut.2020.00120 32974376 PMC7473407

[B128] Sakaguchi Y. Kaimori J. Y. Isaka Y. (2023). Plant-dominant low protein diet: a potential alternative dietary practice for patients with chronic kidney disease. Nutrients 15 (4), 1002. 10.3390/nu15041002 36839360 PMC9964049

[B129] Santoro D. (2008). Low-protein diet and proteinuria. G. Ital. Nefrol. 25 (Suppl. 42), S18–S24. 18828129

[B130] Springmann M. Wiebe K. Mason-D'Croz D. Sulser T. B. Rayner M. Scarborough P. (2018). Health and nutritional aspects of sustainable diet strategies and their association with environmental impacts: a global modelling analysis with country-level detail. Lancet Planet Health 2 (10), e451–e461. 10.1016/S2542-5196(18)30206-7 30318102 PMC6182055

[B131] Storz M. A. (2022). What makes a plant-based diet? a review of current concepts and proposal for a standardized plant-based dietary intervention checklist. Eur. J. Clin. Nutr. 76 (6), 789–800. 10.1038/s41430-021-01023-z 34675405 PMC9187516

[B132] Swiatek L. Jeske J. Miedziaszczyk M. Idasiak-Piechocka I. (2023). The impact of a vegetarian diet on chronic kidney disease (CKD) progression - a systematic review. BMC Nephrol. 24 (1), 168. 10.1186/s12882-023-03233-y 37308813 PMC10259031

[B133] Taccone-Gallucci M. Noce A. Bertucci P. Fabbri C. Manca-di-Villahermosa S. Della-Rovere F. R. (2010). Chronic treatment with statins increases the availability of selenium in the antioxidant defence systems of hemodialysis patients. J. Trace Elem. Med. Biol. 24 (1), 27–30. 10.1016/j.jtemb.2009.06.005 20122576

[B134] Tinti F. Mitterhofer A. P. Umbro I. Nightingale P. Inston N. Ghallab M. (2019). Combined liver-kidney transplantation versus liver transplant alone based on KDIGO stratification of estimated glomerular filtration rate: data from the United Kingdom Transplant registry - a retrospective cohort study. Transpl. Int. 32 (9), 918–932. 10.1111/tri.13413 30793378

[B135] Tonelli M. Wiebe N. Culleton B. House A. Rabbat C. Fok M. (2006). Chronic kidney disease and mortality risk: a systematic review. J. Am. Soc. Nephrol. 17 (7), 2034–2047. 10.1681/ASN.2005101085 16738019

[B136] Tor-Roca A. Garcia-Aloy M. Mattivi F. Llorach R. Andres-Lacueva C. Urpi-Sarda M. (2020). Phytochemicals in legumes: a qualitative reviewed analysis. J. Agric. Food Chem. 68 (47), 13486–13496. 10.1021/acs.jafc.0c04387 33169614

[B137] Turchetti G. Bellelli S. Amato M. Bianchi S. Conti P. Cupisti A. (2017). The social cost of chronic kidney disease in Italy. Eur. J. Health Econ. 18 (7), 847–858. 10.1007/s10198-016-0830-1 27699568 PMC5533856

[B138] United Nations. (2025). Available online at: https://sdgs.un.org/goals (Accessed January 4, 2025).

[B139] van der Weele C. Feindt P. Jan van der Goot A. van Mierlo B. van Boekel M. (2019). Meat alternatives: an integrative comparison. Trends Food Sci. and Technol. 88, 505–512. 10.1016/j.tifs.2019.04.018

[B140] Vanholder R. (2022). Green nephrology. Kidney Dialysis [Online] 2 (3), 454–458. 10.3390/kidneydial2030041

[B141] Wiese G. N. Biruete A. Moorthi R. N. Moe S. M. Lindemann S. R. Hill Gallant K. M. (2021). Plant-based diets, the gut microbiota, and trimethylamine N-oxide production in chronic kidney disease: therapeutic potential and methodological considerations. J. Ren. Nutr. 31 (2), 121–131. 10.1053/j.jrn.2020.04.007 32616440 PMC7770016

[B142] Willett W. Rockstrom J. Loken B. Springmann M. Lang T. Vermeulen S. (2019). Food in the Anthropocene: the EAT-Lancet Commission on healthy diets from sustainable food systems. Lancet 393 (10170), 447–492. 10.1016/S0140-6736(18)31788-4 30660336

[B143] Williams H. Colombi T. Keller T. (2020). The influence of soil management on soil health: an on-farm study in southern Sweden. Geoderma 360, 114010. 10.1016/j.geoderma.2019.114010

[B144] Yin L. Dong X. Liao W. Liu X. Zheng Z. Liu D. (2023). Relationships of beans intake with chronic kidney disease in rural adults: a large-scale cross-sectional study. Front. Nutr. 10, 1117517. 10.3389/fnut.2023.1117517 37081921 PMC10111024

[B145] Zarantonello D. Brunori G. (2023). The role of plant-based diets in preventing and mitigating chronic kidney disease: more light than shadows. J. Clin. Med. 12 (19), 6137. 10.3390/jcm12196137 37834781 PMC10573653

[B146] Zhang N. Cheng Y. Luo R. Chang D. Liu T. Wang Z. (2022). Low-carbohydrate-diet score and mortality in adults with and without chronic kidney disease: results from the third national health and nutrition examination survey. J. Ren. Nutr. 32 (3), 301–311. 10.1053/j.jrn.2021.05.004 34972598

